# Exploring the Neuropharmacological and Antipyretic Effects of *Syzygium grande* (Wight) Walp. Methanolic Leaf Extract: An In Vivo and Computational Study Incorporating GC–MS/MS Analysis

**DOI:** 10.1002/fsn3.71088

**Published:** 2025-10-18

**Authors:** Md. Jahirul Islam Mamun, Sifatul Islam Mizan, Mahathir Mohammad, Md. Liakot Ali, Md. Tanveer Ahsan

**Affiliations:** ^1^ Department of Pharmacy, Faculty of Biological Sciences University of Chittagong Chittagong Bangladesh; ^2^ Department of Chemistry Chittagong University of Engineering and Technology Chattogram Bangladesh

**Keywords:** antidepressant, antipyretic, anxiolytic, GC‐MS/MS, molecular docking, sedative, *Syzygium grande*

## Abstract

*Syzygium grande* (Wight) Walp., commonly known as the sea apple, is valued for its wide range of uses. This study aimed to investigate the pharmacological effects of a methanol extract obtained from the leaves of *S. grande* (MESG) in Swiss albino mice. The extract's anxiolytic potential was assessed using the elevated plus maze (EPM), hole‐board test (HBT), and light–dark test (LDT). Its antidepressant activity was evaluated through the forced swimming test (FST) and tail suspension test (TST). Sedative properties were measured using the open field test (OFT) and hole‐cross test (HCT), while antipyretic activity was determined through a yeast‐induced fever model. To identify active compounds in the extract, GC–MS/MS analysis was conducted. Additionally, computational approaches—including AutoDock Vina, Discovery Studio 2021, PASS online, and ADME/T platforms—were employed for PASS prediction analysis, ADME/T profiling, and molecular docking of various secondary metabolites. The findings indicated that MESG exhibited dose‐dependent anxiolytic, antidepressant, sedative, and antipyretic effects. A 400 mg/kg dose significantly enhanced anxiolytic activity (*p* < 0.001) in all behavioral models. It also moderately reduced immobility in the FST and TST (*p* < 0.01). Furthermore, MESG significantly suppressed locomotor activity in the OFT and HCT (*p* < 0.001), indicating strong sedative action. The same dose effectively lowered rectal temperature in the fever model (*p* < 0.05). Computational docking scores, which ranged from −2.6 to −10 kcal/mol, further supported these biological effects. In conclusion, *S. grande* shows considerable promise as a source of therapeutic agents for treating anxiety, depression, insomnia, and fever.

## Graphical abstract

This study was designed to explore the pharmacological effects of the methanol extract derived from *S. grande* leaves (MESG) on Swiss albino mice. The extract displayed significant anxiolytic, sedative, and antipyretic effects with moderate antidepressant effects. GC–MS/MS analysis identified 64 compounds in MESG, some of which, such as phytol, have been previously reported to possess important neuropharmacological activities.

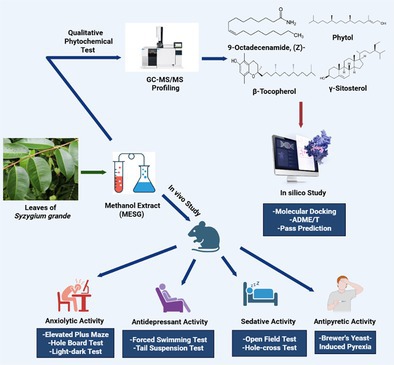

## 1 Introduction

Anxiety disorders are now widely acknowledged as both highly prevalent and chronic, often beginning during adolescence. They affect approximately 18.1% of individuals annually and have a lifetime prevalence of 28.8% (Kessler et al. 2005). These disorders significantly impair quality of life due to associated disabilities (Kasper 1998). Current pharmacological treatments, such as psychotropic medications, are limited by side effects, dietary restrictions, and potential drug interactions (JG 2001). Prolonged use of benzodiazepines at therapeutic doses has been linked to withdrawal symptoms, sedation, and dependence in 20%–100% of patients (Lader et al. 2009). The World Health Organization estimates that mental or behavioral illnesses impact about 450 million individuals globally (Organization 2001). Among these, depression stands out as the most prevalent mental health condition. It is a complex disorder, recognized for its symptomatic, psychological, and biological heterogeneity. Depression is commonly characterized by symptoms such as apathy, loss of energy, slowed thinking and physical activity, and overwhelming feelings of sadness, despair, and, in severe cases, suicidal thoughts or ideation (Santosh et al. 2011). While several synthetic medicines are currently used as reference treatments for clinically depressed patients, they often come with side effects that can hinder the effectiveness of therapy. Dry mouth, exhaustion, gastrointestinal disorders, respiratory disorders, anxiety, agitation, sleepiness, and heart arrhythmias are typical adverse effects. Additionally, the potential for drug–drug interactions further complicates treatment. These challenges have created a growing demand for alternative therapies, particularly those involving medicinal plants, which may offer safer and more sustainable options for managing depression (Dhingra and Sharma 2006). Sedatives and hypnotics are classes of drugs that reduce anxiety and induce a calming effect by promoting sleep and increasing its duration (Katzung et al. 2004). These drugs are frequently recommended to treat a range of mental health issues, such as insomnia and anxiety disorders. However, the prolonged use of currently available sedative‐hypnotic drugs has been associated with significant adverse effects. These can range from impairments in immune, digestive, and respiratory system functions to more severe consequences such as cognitive decline, tolerance, and physical dependence (Dhawan et al. 2003).

The term pyrexia originates from the Greek root pyros, meaning “fire” or “burning heat,” and is essentially the medical equivalent of fever. As such, the terms pyrexia and fever are often used interchangeably. Fever is defined as a regulated increase in body temperature that exceeds the normal range (Estella et al. 2022), typically 36.5°C–37.5°C. This elevation in temperature commonly occurs in response to infections, tissue damage, malignancies, transplant rejection, or other inflammatory conditions (Walter et al. 2016). Notably, up to 75% of critically ill patients experience fever or pyrexia during their illness. Currently available antipyretic medicines, especially nonsteroidal anti‐inflammatory drugs (NSAIDs), have been linked with a range of adverse effects. These include gastrointestinal complications, renal dysfunction, liver damage, and other systemic issues. While selective cyclooxygenase‐2 (COX‐2) inhibitors such as celecoxib, valdecoxib, and rofecoxib were developed to mitigate gastrointestinal side effects, they have been found to pose risks to other organs, including hepatotoxicity (liver damage), nephrotoxicity (kidney damage), neurotoxicity (effects on the brain cortex), and cardiotoxicity (damage to heart muscles) (Aronoff and Neilson 2001). Given these challenges, there is a growing need to explore safer, more effective, and cost‐efficient alternatives for managing pyrexia. Natural products, with their diverse chemical compositions and therapeutic potential, offer a promising avenue for the discovery of novel antipyretic agents.

Computational methods are used in computer‐aided drug design (CADD) to help with drug discovery, identification, and development. It streamlines the procedure, lowers expenses, and expedites the identification of possible medication compounds (Azme et al. 2025). Through computer‐simulated screening, phytochemicals' pharmacological effects can be explained (Parasuraman 2011). The implementation of molecular docking is also crucial for determining the design and preparation of novel medicinal compounds through computer‐aided drug discovery methods. The natural ligand can locate the binding site within the three‐dimensional protein structure and establish a connection through physicochemical interactions via efficient molecular docking. To anticipate the interaction affinities and binding modalities of biological molecules with a particular target receptor, a structure‐based approach using ligand‐receptor molecular docking is typically performed (Guedes et al. 2014).

Natural products have gained growing global attention due to their rich diversity of bioactive compounds and favorable safety profiles with relatively low toxicity compared to synthetic drugs (Hasan et al. 2025). The use of medicinal plants to treat chronic illnesses is widespread worldwide (Adnan et al. 2019). They also play a significant part in the treatment of several illnesses and are a notable source of medicine in Bangladesh's regions (Mollik et al. 2010). Reliable experimental data primarily support the traditional folkloric use of medicinal herbs (Emon et al. 2021). *Syzygium grande* (Wight) Walp., a rare and large evergreen tree, is a distinctive member of the Myrtaceae family and is native to the Southern Western Ghats, India (Mohanan and Sivadasan 2002). It is commonly known as sea apple, jambu ayer laut, jambu laut, or jemba, and holds significant medicinal value in traditional practices (Abdusalam et al. 2022). It has been utilized to address a variety of health conditions, including coughs, piles (hemorrhoids), tooth diseases, dysentery, bronchitis, and diabetes (Rahman et al. 2011). Plants from the Syzygium genus are well documented for their diverse pharmacological properties, with various species exhibiting significant therapeutic potential. For instance, *S. aromaticum* has been shown to possess antifungal effects (Park et al. 2007) (Ayoola et al. 2008) and demonstrates growth‐inhibitory effects against oral pathogens (Cai and Wu 1996). Similarly, *S. cumini* has been reported to exhibit anti‐inflammatory (Chaudhuri et al. 1990), antimicrobial (de Oliveira et al. 2007), and antihyperlipidemic activities (JK 2009), making it a valuable choice in the treatment of inflammatory and metabolic disorders. Additionally, *S. aqueum* has been recognized for its antioxidant activity (Osman et al. 2009), highlighting its potential in combating oxidative stress‐related conditions.

Despite the extensive research on other members of the Syzygium genus, studies on *S. grande* remain limited, particularly regarding its pharmacological properties. To address this gap, the present study evaluated the in vivo anxiolytic, antidepressant, sedative, and antipyretic activity of extracts derived from *S. grande* leaves. Additionally, a computational investigation employing molecular docking was conducted to explore the interactions between the chemical constituents of *S. grande* and receptors associated with these biological activities. This comprehensive approach provides insights into the therapeutic potential of *S. grande* and lays the groundwork for further research into its mechanisms of action and possible applications in drug discovery.

## 2 Materials and Methods

### 2.1 Drugs and Chemicals

In this experiment, we utilized analytical‐grade chemicals and solvents. Square Pharmaceuticals Limited, a Bangladeshi pharmaceutical company, provided fluoxetine, diclofenac sodium, paracetamol, and diazepam. Beximco Pharmaceuticals Limited supplied diethyl ether. The Department of Pharmacy, University of Chittagong, provided the remaining chemicals, all of which were of reagent grade.

### 2.2 Collection and Identification of the Plant

*S. grande* leaves were collected from the botanical garden of the University of Chittagong, Bangladesh. Mr. Mohammad Forkanul Hamid, Department of Fisheries, University of Chittagong, identified the plant under the accession number CU/DP/2025/01.

### 2.3 Preparation of the Plant Extract

To ensure optimal extraction, the gathered leaves (4 kg) were ground into fine powder. Subsequently, the granules were transferred to a sterile glass container for soaking in methanol. For 14 days, the sealed container was stored with occasional shaking. The combination underwent filtration using Whatman filter paper no. 1, and the solvents were removed through evaporation using a rotary evaporator. Dark crude extracts (600 g) were eventually formed by the combination and stored in the refrigerator for subsequent use. Methanol was used as a solvent to prepare *S. grande* leaf extracts, which were referred to as the extract of *S. grande* (MESG).

### 2.4 Acute Oral Toxicity Test

To evaluate acute toxicity, the OECD guidelines (up and down method) were followed to determine the LD_50_ of the test extracts (Rispin et al. 2002). Different concentrations of MESG (100, 200, 400, 1000, 2000, and 4000 mg/kg body weight) were orally administered to groups of animals, with five mice per dose (both sexes). The animals were closely observed for any signs of toxicity or mortality within the first hour postadministration. Subsequently, the rodents were monitored hourly for the next 5–6 h. Over the following 2 weeks, the mice were kept under close observation to detect any delayed adverse effects, behavioral changes, or physiological abnormalities (Hossen, Hossain, et al. 2021).

### 2.5 Gas Chromatography–Mass Spectrometry (GC–MS/MS) Analysis

The MESG was analyzed using a GC‐17A gas chromatograph (Shimadzu) with an Rxi‐5 ms capillary column and a TQ 8040 mass spectrometer. The oven temperature program ranged from 70°C to 220°C, with helium serving as the carrier gas at a flow rate of 0.6 mL/min. The injector and interface temperatures were 260°C and 280°C, respectively. The mass spectrometer scanned a mass range of 40–350 amu (50–550 m/z) using electron impact ionization. One microliter of the sample was injected in splitless mode, with a total run time of 50 min.

### 2.6 Experimental Design and Ethical Approval

Swiss albino mice (20–25 g), both male and female, were sourced from BCSIR, Chattogram, Bangladesh, and housed under controlled conditions (25°C ± 2°C, 45%–55% humidity, 12‐h light–dark cycle). They fasted for 12 h before experimentation. Mice were divided into four groups of five: control (1% Tween 80, 10 mL/kg), test groups (MESG 200 and 400 mg/kg orally), and standard groups (diazepam 1 mg/kg, fluoxetine 25 mg/kg, or paracetamol). All experimental protocols were reviewed and approved by the Institutional Animal Ethics Committee of the University of Chittagong, Bangladesh, under permission number AERB‐CU‐2024‐022. This approval ensures compliance with ethical standards for animal research. Mice were euthanized using ether anesthesia posttrial. All procedures followed ARRIVE guidelines.

### 2.7 Anxiolytic Activity

#### 2.7.1 Elevated Plus Maze Test

This well‐accepted experiment was carried out to assess rodents' anxiolytic properties (Emon et al. 2020). A height of 50 cm separated the elevated plus‐maze from the floor. The animals (*n* = 5) were divided into test, positive control, and negative control groups. Each mouse was placed in the center of the labyrinth and approached the closed arms after being treated with the test extracts (200 and 400 mg/kg), diazepam (1 mg/kg), and vehicle. For 5 min at 0, 30, 60, 90, and 120 min, the amount of time spent in closed and open arms, as well as the total number of open and closed arm entries, was recorded. A sound‐attenuated environment was used to oversee the entire operation.

#### 2.7.2 Hole Board Test

The hole board gadget consisted of 16 evenly spaced holes (3 cm in diameter) in a 40 × 40 × 25 cm wooden chamber. To allow the mice to peek through the holes, the apparatus was built with a platform 25 cm above the ground. Thirty minutes before the start of the experiment, the rodents were given either diazepam (1 mg/kg, i.p.), the vehicle (10 mL/kg, p.o.), or *S. grande* extract (200 and 400 mg/kg, p.o.). Throughout the 5‐min observation period, the quantity and length of each animal's head poking were recorded (Emon et al. 2022).

#### 2.7.3 Light–Dark Box Test

The mice showed a considerable number of transitions to the light compartment in the light–dark box experiment. The number of transitions to the bright compartment (diazepam; 7.0 × 0.58) was 4.67 × 0.33 and 5.67 × 0.67 times higher for the 200 and 400 mg/kg MESG treatments, respectively. Furthermore, the 200 mg/kg MESG treatment resulted in considerable (*p* < 0.01) activity in the light compartment, whereas the 400 mg/kg MESG treatment resulted in the most extended duration spent in the light compartment (Tareq et al. 2020).

### 2.8 Antidepressant Activity

#### 2.8.1 Forced Swimming Test (FST)

Using a previously validated method, the antidepressant activity of MESG in mice was examined through the forced swimming test. A day before the experiment, the mice were acclimatized to the testing apparatus during a preliminary session. The test was conducted in a transparent glass tank measuring 25 × 15 × 25 cm, filled with water to a depth of 15 cm and maintained at a temperature of 25°C ± 1°C. The study included four groups of five mice each. Group I received the vehicle, and Group II was administered fluoxetine (25 mg/kg, i.p.) as a positive control. Groups III and IV were treated orally with MESG at 200 and 400 mg/kg. Following a 30‐min pretreatment period, the mice were placed individually in the tank for 6 min. During this time, their immobility was recorded during the last 4 min of the observation period (S. Hossain et al. 2025).

#### 2.8.2 Tail Suspension Test

The rodents were split up into four groups of five mice each for this modified test. 1% Tween‐80 in saline was administered to Group I as a negative control, and fluoxetine (25 mg/kg, p.o., positive control) was administered to Group II, while *S. grande* extracts at 200 and 400 mg/kg (p.o.) were administered to Groups III and IV. Mice were hung 50 cm above the ground using adhesive tape that was positioned 1 cm from the tips of their tails, 30 min after the treatment. Over 6 min, immobility—which is defined as not moving or hanging silently—was noted (Hossen, Islam, et al. 2021).

### 2.9 Sedative Activity

#### 2.9.1 Open Field Test

The apparatus utilized for this test was surrounded by a wall that was 50 cm high and had a floor area of around 0.5 m^2^ (Gupta et al. 1971). The floor was patterned with alternating black and white squares. At 0, 30, 60, 90, and 120 min post oral treatment, the number of squares explored by the mice was recorded. This included test samples at 200 and 400 mg/kg, the standard group (diazepam), and the control group (saline). The mice's movements were monitored for 3 min using a tally counter.

#### 2.9.2 Hole Cross Test

The experiment was conducted in a chamber made of wooden walls, measuring 30 × 20 × 14 cm, and without a ceiling. A stationary wooden frame is positioned in the center of the space, creating a division into two separate sections. The wooden barrier included a circular aperture measuring 3.5 cm in diameter and 7.5 cm in height. The researchers monitored the frequency of mice passing through the aperture between the two chambers at 0, 30, 60, 90, and 120 min, utilizing a tally counter for 3 min (Eva et al. 2024).

### 2.10 Antipyretic Activity

#### 2.10.1 Brewer's Yeast‐Induced Pyrexia

Fever induced by Brewer's yeast in experimental animals served as the model for evaluating antipyretic effectiveness. A subcutaneous injection of 10 mL of a 20% aqueous yeast solution per kilogram of body weight triggered hyperpyrexia. Before the experiment, the selected animals were fasted for 24 h but had unrestricted access to water. An Ellab thermometer was used to record the initial rectal temperatures of the animals. Eighteen hours after yeast administration, animals exhibiting a rectal temperature increase of 0.3°–0.5°F were chosen for testing antipyretic activity. The extracts were administered orally at a dose of 200 and 400 mg/kg body weight, and their effects were compared to those of paracetamol, which was given orally at 150 mg/kg body weight as a standard medicine. The control group was given only 10 mL of distilled water per kilogram. Following treatment, rectal temperatures were measured hourly for 4 h to assess the antipyretic effects (Zishan et al. 2023).

### 2.11 In Silico Study

#### 2.11.1 Software Tools

The analysis was conducted using the following tools and resources: PubChem, MGL instruments, Swiss pdb‐viewer, AutodockVina, Drug Banking, Discovery Studio Visualizer 2021 (BIOVIA), and Protein Data Bank (PDB).

#### 2.11.2 Validation of the Ligands as Potential Therapeutic Agents

These compounds' physical and molecular properties, as well as pharmacokinetic parameters like ADME/T (absorption, distribution, metabolism, excretion, and toxicity), significantly impact the choice of these compounds as treatment possibilities. The PubChem database (pubchem.ncbi.nlm.nih.gov) was used to obtain selected compounds from the GC–MS/MS of MESG. The pKCSM online tool (http://biosig.unimelb.edu.au/pkcsm/), which was accessed on September 11, 2024, was used to confirm the listed compounds' potential as ligands against therapeutic targets (Pires et al. 2015). The compounds were then evaluated for drug potential utilizing Lipinski's rule of five using the SwissADME web server (Daina et al. 2017).

#### 2.11.3 Protein Preparation and Active Site Determination

The RCSB protein data bank provided the crystal structures for the target protein, human monoamine oxidase (PDB: 2Z5X) (Ali et al. 2025), human serotonin transporter (PDB: 5I6X) (Mohammad, Chowdhury, et al. 2025), human GABA_A_ receptor alpha1‐beta2‐gamma2 subtype (PDB: 6X3T) (Mohammad, Rasel, et al. 2025), and microsomal prostaglandin E synthase 1 (mPGES‐1) (PDB: 4YK5) (Mohammad, Mamun, et al. 2025). The active site of the enzyme was found using the previously published data by Kurumbail et al. (1996). Swiss‐PdbViewer (v4.1) and the BIOVIA Discovery Studio 4.5 Client were utilized to carry out the required cleaning and preparations, including the elimination of heteroatoms, cofactors, and water. Following the introduction of hydrogen atoms into the target protein's structure, the PyRx virtual screening tool and the MMFF94 force field were employed to optimize the protein. The target protein was kept in pdb format to make docking investigations simpler.

#### 2.11.4 Molecular Docking and Postdocking Analysis

Docking calculations were carried out with AutoDock, version 4.2, and PyRx 0.3 (http://pyrx.scripps.edu) (accessed on September 11, 2024) (Saddala et al. 2013). Using AutoGrid, a grid box measuring X: 54.057; Y: 32.4307; Z: 39.9567 Å points was constructed, with a grid spacing of 0.375 Å. PyMOL was used to analyze the results of the docking. These tools can help identify whether the type of interaction—hydrogen bond, π‐π, or cation‐π interactions, for example—contributes to ligand binding. PyMOL (Protein–ligand docking: Current state and future challenges) was utilized to gather additional information on the interaction between ligands and receptors.

#### 2.11.5 ADME/T Study

Since toxicity is a significant concern in the development of new drugs, the toxicological properties and ADME/T study of the recently identified compounds were evaluated using the AdmetSAR online tool (http://lmmd.ecust.edu.cn/admetsar1/predict/). Chemicals from that list were screened using SwissADME (Mohammad, Jahirul Islam, et al. 2025) and the Lipinski rule of five (Ali et al. 2024).

#### 2.11.6 PASS Prediction

The PASS (Prediction of Activity Spectra for Substances) program was used to analyze the structures of eighteen compounds from MESG for their potential anxiolytic, antidepressant, sedative, and antipyretic activities. The program employs a structure–activity relationship (SAR) approach to predict the biological activity spectrum of each compound, categorizing it as either probable activity (Pa) or probable inactivity (Pi). A compound is considered experimentally active if Pa > Pi, with values from 0.000 to 1.000. A Pa value > 0.7 indicates high medicinal activity, suggesting that such compounds are highly likely to exhibit significant therapeutic effects. This computational prediction aids in identifying promising candidates for further experimental validation (Jiko et al. 2024).

### 2.12 Statistical Analysis

The findings were displayed as Mean ± SEM. The statistical software “Statistical Package for the Social Sciences” (SPSS, Version 16.0, IBM Corporation, New York) was used to conduct the statistical analysis. “GraphPad Prism 8 and MS Excel 2024” were used for drawing the graph. For drawing chemical structures, “ChemDraw Ultra 12.0” was used. A post hoc Dunnett test was utilized for comparisons following a one‐way analysis of variance (ANOVA). The significance levels were established using the following criteria: *p < 0.05, **p < 0.01, and ***p < 0.001. Statistical significance is seen in these statistics when compared to the study group.

## 3 Results

### 3.1 Acute Oral Toxicity Test

The first step involved the purification of the compound, beginning with the harvesting of the plant in its natural environment. No signs of toxicity or mortality were observed at any of the tested doses during the toxicity test. Daily food and water intake remained within normal limits, indicating no adverse effects on the animals' health or behavior (Table 1). The LD_50_ of MESG was determined to be greater than 4000 mg/kg, confirming its safety and stability at oral doses up to 4000 mg/kg body weight. Based on these findings, doses of 200 and 400 mg/kg were selected for further pharmacological evaluation to ensure efficacy and safety while remaining below the threshold of adverse effects.

**TABLE 1:** General appearance and behavioral observations for control and treated groups during the acute toxicity study.

Observation	Control group		2000 mg/kg		4000 mg/kg	
	5 h	24 h	5 h	24 h	5 h	24 h
Skin and fur	Normal	Normal	Normal	Normal	Normal	Normal
Eyes	Normal	Normal	Normal	Normal	Normal	Normal
Mucous membrane	Normal	Normal	Normal	Normal	Normal	Normal
Behavioral patterns	Normal	Normal	Normal	Normal	Increased heartbeat	Normal
Salivation	Normal	Normal	N.O.	N.O.	N.O.	N.O.
Lethargy	Normal	Normal	N.O.	N.O.	Observed	N.O.
Sleep	Normal	Normal	N.O.	N.O.	N.O.	N.O.
Diarrheal	Normal	Normal	N.O.	N.O.	N.O.	Observed
Coma	N.O.	N.O.	N.O.	N.O.	N.O.	N.O.
Tremors	N.O.	N.O.	N.O.	N.O.	N.O.	N.O.

Abbreviation: N.O., not observed.

### 3.2 GC–MS/MS Profiling

**FIGURE 1:**
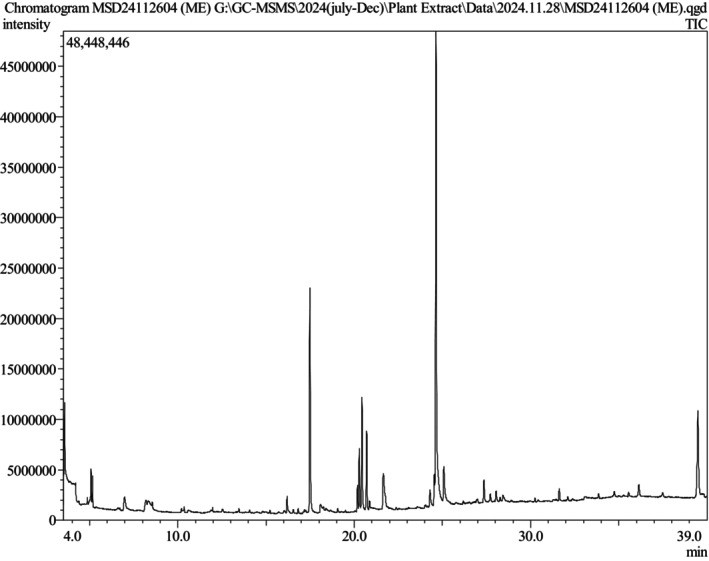
*S. grande* leaves methanolic extract's GC–MS/MS chromatogram.

**FIGURE 2:**
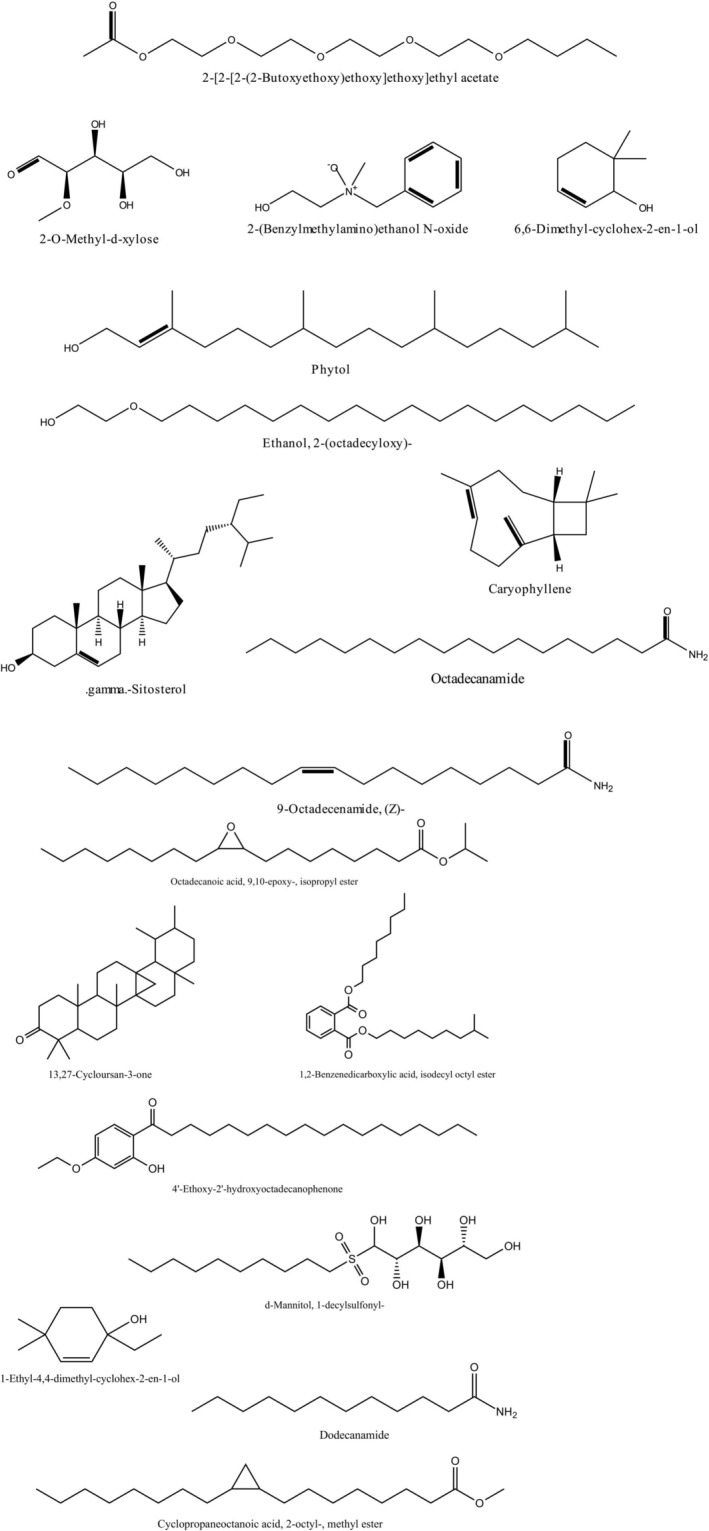
GC–MS/MS compounds of the methanolic leaves extract of *S. grande* .

The GC–MS/MS analysis of the phytoconstituent‐rich extract MESG identified a total of 64 compounds, each exhibiting unique phytochemical activity. The predominant compound in the extract was 9‐Octadecenamide (Z‐), accounting for 28.98% of the total composition. Other significant compounds include Hexadecanoic acid, methyl ester (9.79%), 2‐(Benzylmethylamino)ethanol N‐oxide (7.07%), gamma‐sitosterol (6.51%), and Phytol (5.08%). These compounds are known for their diverse biological activities, contributing to the overall therapeutic potential of the extract. The chromatograms obtained from the analysis are presented in Figure 1. At the same time, Table 2 provides detailed information about the identified compounds, including their molecular formulas, molecular weights, retention times (RTs), m/z values, and peak area percentages, along with chemical structures (Figure 2). This comprehensive characterization highlights the rich phytochemical diversity of MESG and lays the foundation for further exploration of its pharmacological properties.

**TABLE 2:** GC–MS/MS compounds identified in MESG. MESG, methanolic extract of *S. grande* .

Sl No.	Compound name	Molecular formula	Molecular weight (g/mol)	R. time	Area %	*m*/*z*
1	9‐Octadecenamide, (Z)—	C_18_H_35_NO	281.5	24.632	28.98	59
2	Hexadecanoic acid, methyl ester	C_17_H_34_O_2_	270.5	17.483	9.79	74
3	2‐(Benzylmethylamino)ethanol N‐oxide	C_10_H_15_NO_2_	181.23	3.576	7.07	91
4	.gamma.‐Sitosterol	C_29_H_50_O	414.7	39.478	6.51	43
5	Phytol	C_20_H_40_O	296.5	20.436	5.08	71
6	Hexadecanamide	C_16_H_33_NO	255.44	21.648	3.55	59
7	Methyl stearate	C_19_H_38_O_2_	298.5	20.704	3.33	74
8	Octadecanamide	C_18_H_37_NO	283.5	25.08	2.52	59
9	8,11,14‐Docosatrienoic acid, methyl ester	C_23_H_40_O_2_	348.6	20.287	2.46	55
10	3,3‐Dimethoxy‐2‐butanone	C_6_H_12_O_3_	132.16	5.085	1.6	89
11	9,11‐Octadecadienoic acid, methyl ester, (E,E)—	C_19_H_34_O_2_	294.5	24.539	1.32	67
12	Hexacosane‐4,6‐dione	C_26_H_50_O_2_	394.7	24.291	1.31	71
13	Undecane	C_11_H_24_	156.31	6.983	1.16	57
14	1,2‐Benzenedicarboxylic acid, isodecyl octyl ester	C_26_H_42_O_4_	418.6	27.352	1.1	149
15	Pentadecanoic acid	C_15_H_30_O_2_	242.4	18.085	0.84	73
16	4,6‐Dioxatetradecane	C_12_H_26_O_2_	202.33	8.37	0.79	73
17	Neophytadiene	C_20_H_38_	278.5	16.192	0.78	68
18	Vitamin E	—	—	36.135	0.76	165
19	Oxazolidin‐2‐one	C_3_H_5_NO_2_	87.08	4.03	0.75	89
20	Di‐sec‐Butyl ether	C_8_H_18_O	130.229	5.166	0.74	103
21	2,2‐Dimethoxybutane	C_6_H_14_O_2_	118.17	4.207	0.73	87
22	Squalene	C_30_H_50_	410.7	31.62	0.58	69
23	3‐Nitropropanoic acid	C_3_H_5_NO_4_	119.08	4.118	0.57	32
24	4′‐Ethoxy‐2′‐hydroxyoctadecanophenone	C_26_H_44_O_3_	404.6	28.042	0.54	180
25	2‐O‐Methyl‐d‐xylose	C_6_H_12_O_5_	164.16	3.915	0.51	87
26	2‐[2‐[2‐(2‐Butoxyethoxy) ethoxy]ethoxy]ethyl acetate	C_14_H_28_O_6_	292.37	3.955	0.49	87
27	1,2‐Butanediol, 3,3‐dimethyl—	C_6_H_14_O_2_	118.17	4.15	0.48	28
28	2‐Octyldodecyl butyrate	C_24_H_48_O_2_	368.6	28.429	0.48	281
29	6,6‐Dimethyl‐cyclohex‐2‐en‐1‐ol	C_8_H_14_O	126.2	27.713	0.43	70
30	Hydroxyacetic acid, hydrazide	C_2_H_6_N_2_O_2_	90.08	3.52	0.37	118.17 g28
31	Dodecanamide	C_12_H_25_NO	199.33	18.403	0.37	59
32	1,3‐Dioxane	C_4_H_8_O_2_	88.11	3.99	0.34	87
33	1‐Ethyl‐4,4‐dimethyl‐cyclohex‐2‐en‐1‐ol	C_10_H_18_O	154.25	26.982	0.34	125
34	Cyclopropaneoctanoic acid, 2‐octyl‐, methyl ester	C_20_H_38_O_2_	310.5	20.868	0.31	87
35	13,27‐Cycloursan‐3‐one	C_30_H_48_O	424.7	39.703	0.29	95
36	4‐Bromobutanoic acid, pentadecyl ester	C_19_H_37_BrO_2_	377.4	33.073	0.26	223
37	.beta.‐Tocopherol	C_28_H_48_O_2_	416.7	34.745	0.25	151
38	Emylcamate	C_7_H_15_NO_2_	145.2	8.505	0.22	73
39	Propanedioic acid, (1,2‐dimethylpropylidene)‐, dimethyl ester	C_10_H_16_O_4_	200.23	10.206	0.2	164
40	3,7,11,15‐Tetramethyl‐2‐hexadecen‐1‐ol	C_20_H_40_O	296.5	16.817	0.2	81
41	Methyl 18‐methylnonadecanoate	C_21_H_42_O_2_	326.6	24.047	0.17	74
42	Ethanol, 2‐(octadecyloxy)—	C_20_H_42_O_2_	314.5	26.517	0.16	57
43	Nonyl tetracosyl ether	C_33_H_68_O	480.9	26.66	0.16	71
44	Trieicosane, 1‐bromo‐11‐docosenyliden—	C_35_H_69_Br	569.8	31.319	0.16	57
45	2‐Heptadecanone	C_17_H_34_O	254.5	17.177	0.15	58
46	Hexadecanoic acid, 15‐methyl‐, methyl ester	C_18_H_36_O_2_	284.5	19.062	0.14	74
47	Heptadecanal	C_17_H_34_O	254.5	16.065	0.13	57
48	2‐Methyltetracosane	C_25_H_52_	352.7	18.648	0.13	57
49	Pentadecane, 8‐hexyl—	C_21_H_44_	296.6	25.34	0.13	57
50	1,1,3,3‐Tetraallyl‐1,3‐disilacyclobutane	C_14_H_24_Si_2_	248.51	31.443	0.13	71
51	Octadecanoic acid, 9,10‐epoxy‐, isopropyl ester	C_21_H_40_O_3_	340.5	32.374	0.12	207
52	Caryophyllene	C_15_H_24_	204.35	11.845	0.11	91
53	2,4‐Di‐tert‐butylphenol	C_14_H_22_O	206.32	12.525	0.09	191
54	Methyl tetradecanoate	C_15_H_30_O_2_	242.4	14.81	0.09	74
55	Triacontane, 1‐bromo—	C_30_H_61_Br	501.7	26.834	0.09	57
56	2,6‐Dihydroxybenzoic acid, 3TMS derivative	C_16_H_30_O_4_Si_3_	370.66	16.003	0.08	73
57	Nonadecanoic acid, methyl ester	C_20_H_40_O_2_	312.5	22.375	0.08	74
58	Butanoic acid, 2‐methyl‐, methyl ester	C_6_H_12_O_2_	116.16	4.388	0.06	91
59	Heptadecanoic acid, 7‐iodo‐, methyl ester	C_18_H_35_IO_2_	410.4	30.421	0.06	57
60	Rhamnitol, 1‐O‐decyl—	C_16_H_34_O_5_	306.44	17.115	0.05	73
61	9‐Octadecenenitrile, (Z)—	C_18_H_33_N	263.5	20.035	0.05	55
62	9‐Octadecene, 1,1‐dimethoxy‐, (Z)—	C_20_H_40_O_2_	312.5	32.03	0.05	28
63	2‐Hexenoic acid, 5‐hydroxy‐3,4,4‐trimethyl‐, (E)—	C_9_H_16_O_3_	172.22	31.5	0.04	28
64	d‐Mannitol, 1‐decylsulfonyl—	C_16_H_34_O_7_S	370.5	34.67	0.04	207

### 3.3 Anxiolytic Activity

#### 3.3.1 Elevated Plus Maze Test

**FIGURE 3:**
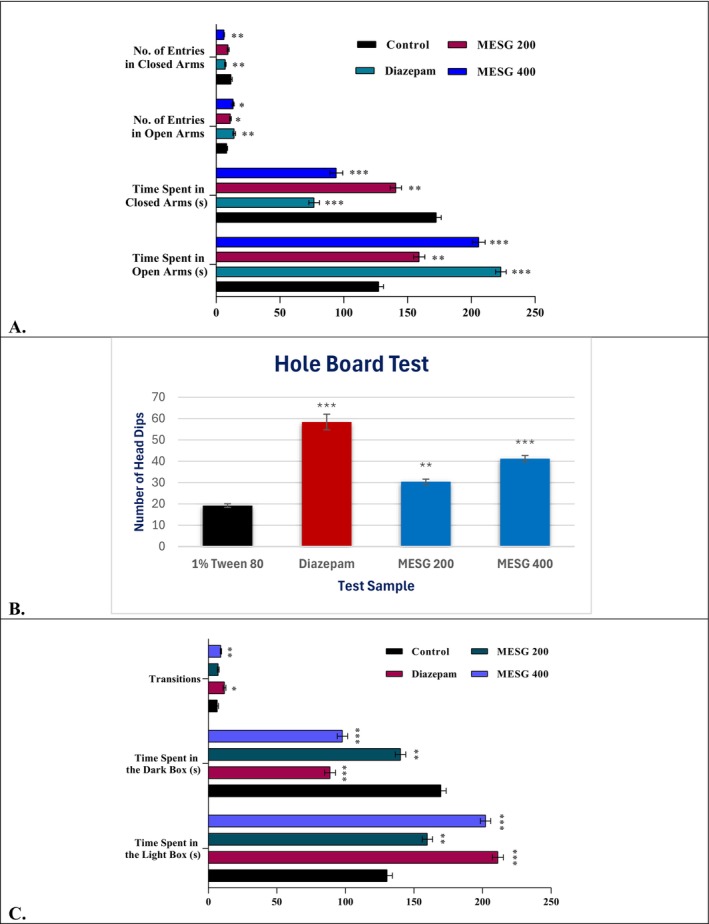
Anxiolytic effects of MESG on tested mice through. (A) Elevated Plus Maze Test (B), Hole Board Test, and (C) Light Dark Box Test. MESG, methanolic extract of *S. grande* . All values are presented as Mean ± SEM, and statistical analysis was performed using One‐Way Analysis of Variance (ANOVA). Subsequently, *n* = 5 is employed for Dunnett's multiple comparison test, with *p < 0.05, **p < 0.01, and ***p < 0.001 in comparison to the control group.

MESG‐treated mice spent significantly more time in the open arms of the Elevated Plus Maze (EPM) compared to controls, with the 400 mg/kg group spending 205.8 ± 5.03 s (*p* < 0.001) versus 127.4 ± 3.79 s for controls. Diazepam‐treated mice spent the most time in the open arms (223.2 ± 4.12 s, *p* < 0.001). While MESG‐treated mice spent more time in the closed arms than the diazepam group, they exhibited reduced anxiety‐like behavior compared to controls, as evidenced by increased open‐arm exploration. These findings indicate that MESG possesses significant anxiolytic activity (Table 3 and Figure 3A).

**TABLE 3:** Evaluation of anxiolytic activity of MESG through Elevated Plus Maze Test. MESG, Methanolic Extract of *S. grande* .

Test samples	Dose (mg/kg)	Time spent in open arms (sec)	Time spent in closed arms (sec)	No. of entries in open‐arm	No. of entries in closed‐arm
Control	10	127.4 ± 3.79	172.6 ± 3.79	8.4 ± 0.51	11.6 ± 0.93
Diazepam	1	223.2 ± 4.12***	76.8 ± 4.12***	14.2 ± 1.02**	7.2 ± 0.58**
MESG	200	159.2 ± 4.43**	140.8 ± 4.43**	11.2 ± 0.73*	9.4 ± 0.75
MESG	400	205.8 ± 5.03***	94.2 ± 5.03***	13.4 ± 0.51*	6.2 ± 0.37**

*Note:* All values are presented as Mean ± SEM, and statistical analysis was performed using One‐Way Analysis of Variance (ANOVA). Subsequently, *n* = 5 is employed for Dunnett's multiple comparison test, with *p < 0.05, **p < 0.01, and ***p < 0.001 in comparison to the control group.

#### 3.3.2 Hole Board Test

In the hole board test, MESG at 400 mg/kg significantly increased head dipping behavior (41.2 ± 1.46, *p* < 0.001) compared to the control group (19.2 ± 0.86). Diazepam, as the positive control, showed an even higher increase (58.4 ± 3.67, *p* < 0.001). The increased head‐dipping frequency with MESG indicates enhanced exploratory behavior and reduced anxiety‐like behavior. These results suggest that MESG exhibits vigorous anxiolytic activity, though slightly less potent than diazepam (Table 4 and Figure 3B).

**TABLE 4:** Evaluation of anxiolytic activity of MESG using hole board test. MESG, methanolic extract of *S. grande* .

Test sample	Dose (mg/kg)	Number of head dipping					Mean ± SEM
		M1	M2	M3	M4	M5	
1% Tween 80	0.10 mL/10 g	17	19	22	18	20	19.2 ± 0.86
Diazepam	1	54	59	70	61	48	58.4 **±** 3.67***
MESG	200	34	29	32	30	27	30.4 **±** 1.21**
MESG	400	40	39	43	38	46	41.2 ± 1.46***

*Note:* All values are presented as Mean ± SEM, and statistical analysis was performed using One‐Way Analysis of Variance (ANOVA). Subsequently, *n* = 5 is employed for Dunnett's multiple comparison test, with *p < 0.05, **p < 0.01, and ***p < 0.001 in comparison to the control group.

#### 3.3.3 Light–Dark Box Test

In the light–dark test, mice treated with MESG at 400 mg/kg spent significantly more time in the light box (202.2 ± 3.77 s; *p* < 0.001) compared to controls (130.4 ± 3.85 s). The diazepam‐treated group showed even greater anxiolytic activity (211.2 ± 3.98 s; *p* < 0.001). Both doses of MESG increased time spent in the light box and reduced time in the dark box, indicating reduced anxiety‐like behavior. Additionally, MESG‐treated mice made more transitions between compartments than controls, further confirming its anxiolytic properties (Figure 3C).

### 3.4 Antidepressant Activity

#### 3.4.1 Forced Swimming Test

**FIGURE 4:**
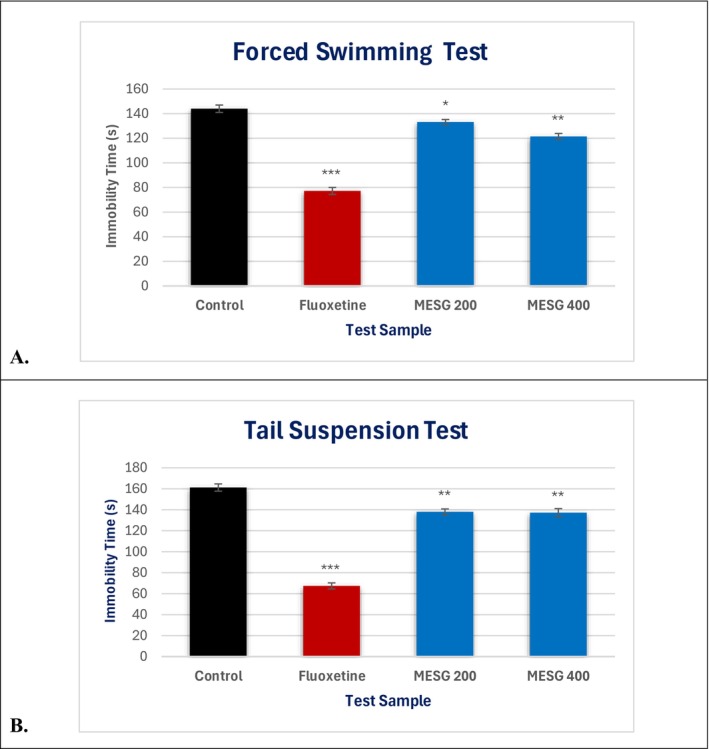
Antidepressant effects of MESG on tested mice through, (A) Forced Swimming Test and (B) Tail Suspension Test. MESG, methanolic extract of *S. grande* . All values are presented as Mean ± SEM, and statistical analysis was performed using One‐Way Analysis of Variance (ANOVA). Subsequently, *n* = 5 is employed for Dunnett's multiple comparison test, with *p < 0.05, **p < 0.01, and ***p < 0.001 in comparison to the control group.

In this test, MESG demonstrated mild antidepressant activity compared to the control group. At doses of 200 mg/kg and 400 mg/kg, MESG significantly reduced the immobility time of mice to 133.2 ± 1.98 s and 121.4 ± 2.46 s, respectively. However, the reduction in immobility time was more pronounced with the standard antidepressant fluoxetine, which decreased immobility time to 77.2 ± 2.78 s (*p* < 0.001) compared to the control group (144 ± 2.98 s) (Table 5 and Figure 4A).

**TABLE 5:** Evaluation of antidepressant activity of methanolic extract of *S. grande* (MESG) through the forced swimming test.

Sample	Dose (mg/kg)	Immobility time					
		S‐1	S‐2	S‐3	S‐4	S‐5	Mean ± SEM
Control	10	140	137	154	142	147	144 ± 2.98
Fluoxetine	25	84	73	82	78	69	77.2 ± 2.78***
MESG	200	130	139	128	136	133	133.2 ± 1.98*
MESG	400	129	116	120	117	125	121.4 ± 2.46**

*Note:* All values are presented as Mean ± SEM, and statistical analysis was performed using One‐Way Analysis of Variance (ANOVA). Subsequently, *n* = 5 is employed for Dunnett's multiple comparison test, with *p < 0.05, **p < 0.01, and ***p < 0.001 in comparison to the control group.

#### 3.4.2 Tail Suspension Test

MESG exhibited moderate antidepressant activity in the study, as evidenced by the reduction in immobility time at both doses. At 200 mg/kg and 400 mg/kg, MESG decreased immobility time to 138 ± 2.77 s and 137.2 ± 3.88 s, respectively, compared to the control group (161.2 ± 3.48 s). However, the standard drug fluoxetine demonstrated significantly greater efficacy, reducing immobility time to 67.4 ± 2.87 s (*p* < 0.001). These findings suggest that while MESG moderately alleviates depressive‐like behavior, its effects are less potent than those of fluoxetine (Figure 4B).

### 3.5 Sedative Activity

#### 3.5.1 Open Field Test

**FIGURE 5:**
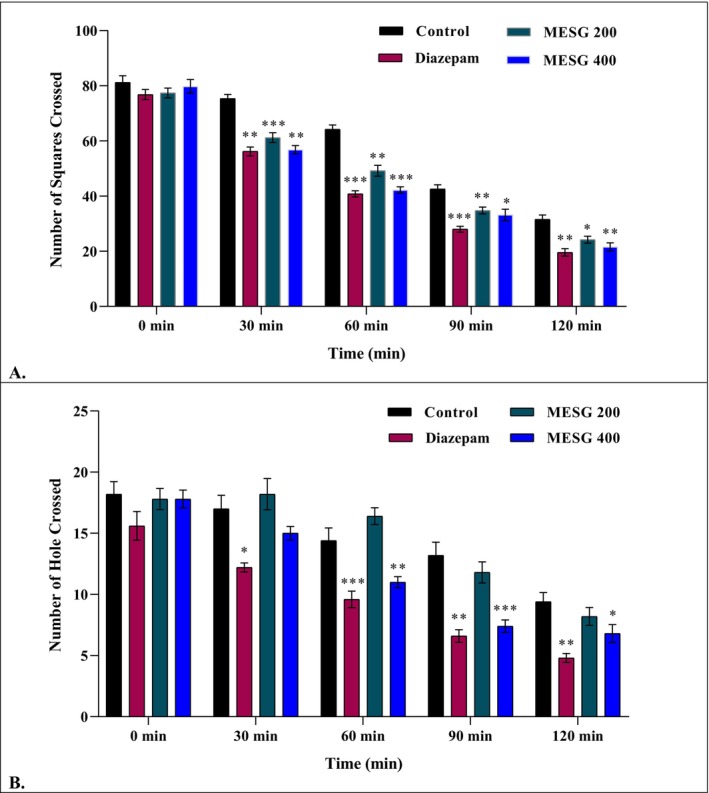
Sedative effects of MESG on tested mice through, (A) Open field test and (B) Hole cross test. MESG, methanolic extract of *S. grande* . All values are presented as Mean ± SEM, and statistical analysis was performed using One‐Way Analysis of Variance (ANOVA). Subsequently, *n* = 5 is employed for Dunnett's multiple comparison test, with *p < 0.05, **p < 0.01, and ***p < 0.001 in comparison to the control group.

In the open field test, MESG‐treated mice (200 mg/kg and 400 mg/kg) showed a significant dose‐dependent reduction in crossed squares over time compared to controls. At 30 min, the 200 mg/kg and 400 mg/kg groups crossed 61.2 ± 1.77 and 42.2 ± 1.16 squares, respectively, versus 75.4 ± 1.50 for controls. At 60 min, the 400 mg/kg group crossed 42.2 ± 1.16 squares, compared to 64.2 ± 1.59 for controls. Diazepam further reduced locomotor activity (e.g., 40.8 ± 1.11 at 60 min, *p* < 0.001). The progressive decline in movement indicates that MESG exhibits notable sedative activity (Table 6 and Figure 5A).

**TABLE 6:** Evaluation of sedative activity of Methanolic Extract of *S. grande* (MESG) through the open field test.

Treatment dose (mg/kg)	Number of squares crossed				
	0 min	30 min	60 min	90 min	120 min
Control	81.2 ± 2.46	75.4 ± 1.50	64.2 ± 1.59	42.6 ± 1.50	31.6 ± 1.57
Diazepam	76.8 ± 1.83	56.2 ± 1.59**	40.8 ± 1.11***	28 ± 1.10***	19.6 ± 1.36**
MESG 200	77.4 ± 1.78	61.2 ± 1.77***	49.2 ± 1.96**	34.8 ± 1.24**	24.2 ± 1.28*
MESG 400	79.8 ± 2.48	56.8 ± 1.50**	42.2 ± 1.16***	33.2 ± 2.06*	21.6 ± 1.44**

*Note:* All values are presented as Mean ± SEM, and statistical analysis was performed using One‐Way Analysis of Variance (ANOVA). Subsequently, *n* = 5 is employed for Dunnett's multiple comparison test, with *p < 0.05, **p < 0.01, and ***p < 0.001 in comparison to the control group.

#### 3.5.2 Hole Cross Test

In the hole cross test, MESG exhibited significant sedative effects in a dose‐dependent manner. At 90 min, the 400 mg/kg group showed a marked reduction in mouse movement (7.4 ± 0.51, *p* < 0.001) compared to the control group (13.2 ± 1.07). Diazepam demonstrated more substantial sedative effects, with a significant reduction in movement as early as 60 min (9.6 ± 0.68). These results confirm the dose‐dependent sedative activity of MESG, although its onset and potency were less pronounced than diazepam, highlighting its potential as a natural sedative agent (Figure 5B).

### 3.6 Antipyretic Activity

#### 3.6.1 Brewer's Yeast‐Induced Pyrexia Method

**FIGURE 6:**
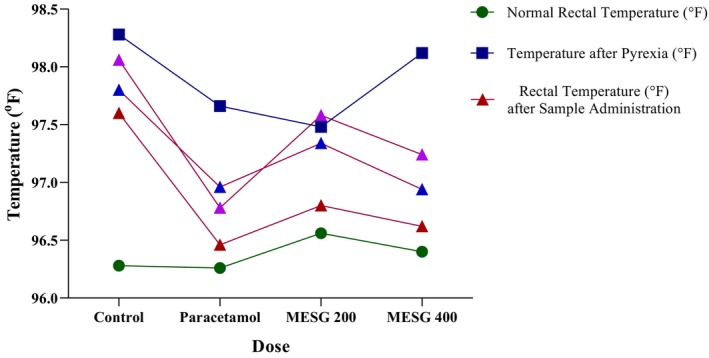
Antipyretic effects of MESG on tested mice using the Brewer's Yeast‐induced Pyrexia method. MESG, methanolic extract of *S. grande* .

In this test, MESG exhibited a modest antipyretic effect compared to the control group. Pyrexia was induced in the mice by administering a yeast suspension subcutaneously. At the 400 mg/kg dose, MESG notably reduced rectal temperature to 96.62° ± 0.29°F after 180 min of treatment, compared to the control group, which had a rectal temperature of 97.6° ± 0.25°F. The standard drug paracetamol demonstrated a more significant antipyretic effect, lowering rectal temperature to 96.46°± 0.19°F (*p* < 0.05) at the same time point. These results indicate that MESG exhibits notable antipyretic activity, comparable to that of the standard paracetamol (Table 7 and Figure 6).

**TABLE 7:** Evaluation of antipyretic activity of Methanolic Extract of *S. grande* (MESG) through Brewer's Yeast–induced Pyrexia Method.

Treatment	Normal rectal temperature (°F)	Temperature after Pyrexia (°F)	Rectal temperature (°F) after the sample administration		
			60 min	120 min	180 min
Control	96.28 ± 0.26	98.28 ± 0.17	98.06 ± 0.28	97.8 ± 0.23	97.6 ± 0.25
Paracetamol	96.26 ± 0.23	97.66 ± 0.18	96.78 ± 0.35*	96.96 ± 0.19*	96.46 ± 0.19*
MESG 200	96.56 ± 0.29	97.48 ± 0.12**	97.58 ± 0.12**	97.34 ± 0.27	96.8 ± 0.17*
MESG 400	96.4 ± 0.38	98.12 ± 0.2	97.24 ± 0.2	96.94 ± 0.27*	96.62 ± 0.29*

*Note:* All values are presented as Mean ± SEM, and statistical analysis was performed using One‐Way Analysis of Variance (ANOVA). Subsequently, *n* = 5 is employed for Dunnett's multiple comparison test, with *p < 0.05, **p < 0.01, and ***p < 0.001 in comparison to the control group.

### 3.7 In Silico Study

#### 3.7.1 ADME/T Study

The study confirms that each molecule meets Lipinski's criteria, making it suitable for oral administration. Additionally, the toxicological profiles of the 18 compounds were predicted using two online tools: pKCSM (http://biosig.unimelb.edu.au/pkcsm/) and admetSAR (http://lmmd.ecust.edu.cn/admetsar2/). The analysis reveals that the compounds are neither toxic nor carcinogenic (Table 8).

**TABLE 8:** In silico AdmetSAR and drug likeliness study of selected compounds of Methanolic Extract of *S. grande* .

Compounds name	Absorption		Distribution		Metabolism	Excretion	Toxicity		Drug likeliness	Bioavailability
	Water solubility (log mol/L)	Intestinal absorption (Human) (% Absorbed)	VDss (Human) (log L/kg)	BBB permeability (log BB)	CYP3A4 substrate	Total clearance (log mL/min/kg)	AMES toxicity	Hepatotoxicity		
2‐O‐Methyl‐d‐xylose	0.306	52.067	−0.506	−0.959	No	0.905	No	No	Yes	0.55
2‐(Benzylmethylamino)ethanol N‐oxide	−1.277	80.544	0.03	−0.093	No	0.542	No	No	Yes	0.55
2‐[2‐[2‐(2‐Butoxyethoxy)ethoxy]ethoxy]ethyl acetate	−2.078	98.528	−0.322	−0.023	No	0.887	No	No	Yes	0.55
Phytol	−7.535	90.643	0.385	0.793	Yes	1.686	No	No	Yes	0.55
9‐Octadecenamide, (Z)—	−7.074	90.218	0.281	−0.389	Yes	1.959	No	No	Yes	0.55
6,6‐Dimethyl‐cyclohex‐2‐en‐1‐ol	−1.182	94.746	0.132	0.135	No	0.169	No	No	Yes	0.55
Caryophyllene	−5.555	94.845	0.652	0.733	No	1.088	No	No	Yes	0.55
.gamma.‐Sitosterol	−6.773	94.46	0.193	0.781	Yes	Yes	No	No	Yes	0.55
Octadecanamide	−7.17	89.712	0.306	−0.416	Yes	1.906	No	No	Yes	0.55
Ethanol, 2‐(octadecyloxy)—	−7.213	89.479	0.347	0.842	Yes	2.07	No	No	Yes	0.55
1‐Ethyl‐4,4‐dimethyl‐cyclohex‐2‐en‐1‐ol	−2.039	94.183	0.207	0.301	No	1.204	No	No	Yes	0.55
1,2‐Benzenedicarboxylic acid, isodecyl octyl ester	−6.757	90.622	0.26	−0.313	Yes	1.811	No	No	Yes	0.55
4′‐Ethoxy‐2′‐hydroxyoctadecanophenone	−7.386	88.101	0.465	−0.614	Yes	1.69	No	No	Yes	0.55
Octadecanoic acid, 9,10‐epoxy‐, isopropyl ester	−5.308	92.842	−0.053	−0.134	Yes	1.701	No	No	Yes	0.55
d‐Mannitol, 1‐decylsulfonyl—	−2.239	39.665	−0.854	−1.678	No	2.135	No	No	Yes	0.55
13,27‐Cycloursan‐3‐one	−5.231	100	−0.197	0.776	No	−0.073	No	No	Yes	0.55
Dodecanamide	−4.596	91.773	0.248	−0.164	No	1.697	No	No	Yes	0.55
Cyclopropaneoctanoic acid, 2‐octyl‐, methyl ester	−7.458	92.608	0.297	0.815	Yes	1.645	No	No	Yes	0.55

#### 3.7.2 Molecular Docking Study

**FIGURE 7:**
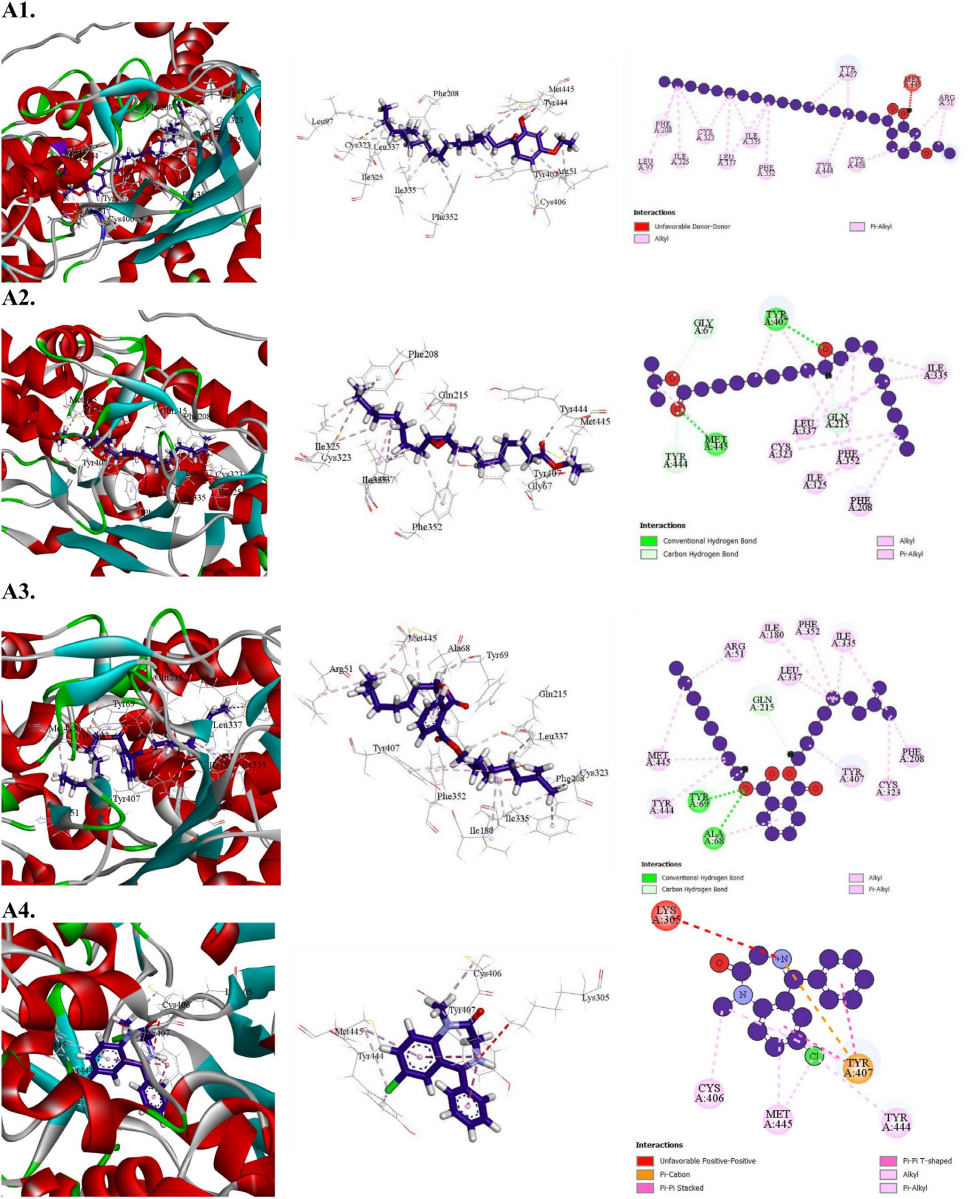
Molecular docking interaction of compounds against the human monoamine oxidase (PDB: 2Z5X): (A1) 4′‐Ethoxy‐2′‐hydroxyoctadecanophenone, (A2) Octadecanoic acid, 9,10‐epoxy‐, isopropyl ester, (A3) 1,2‐Benzenedicarboxylic acid, isodecyl octyl ester, (A4) Diazepam (Standard).

**FIGURE 8:**
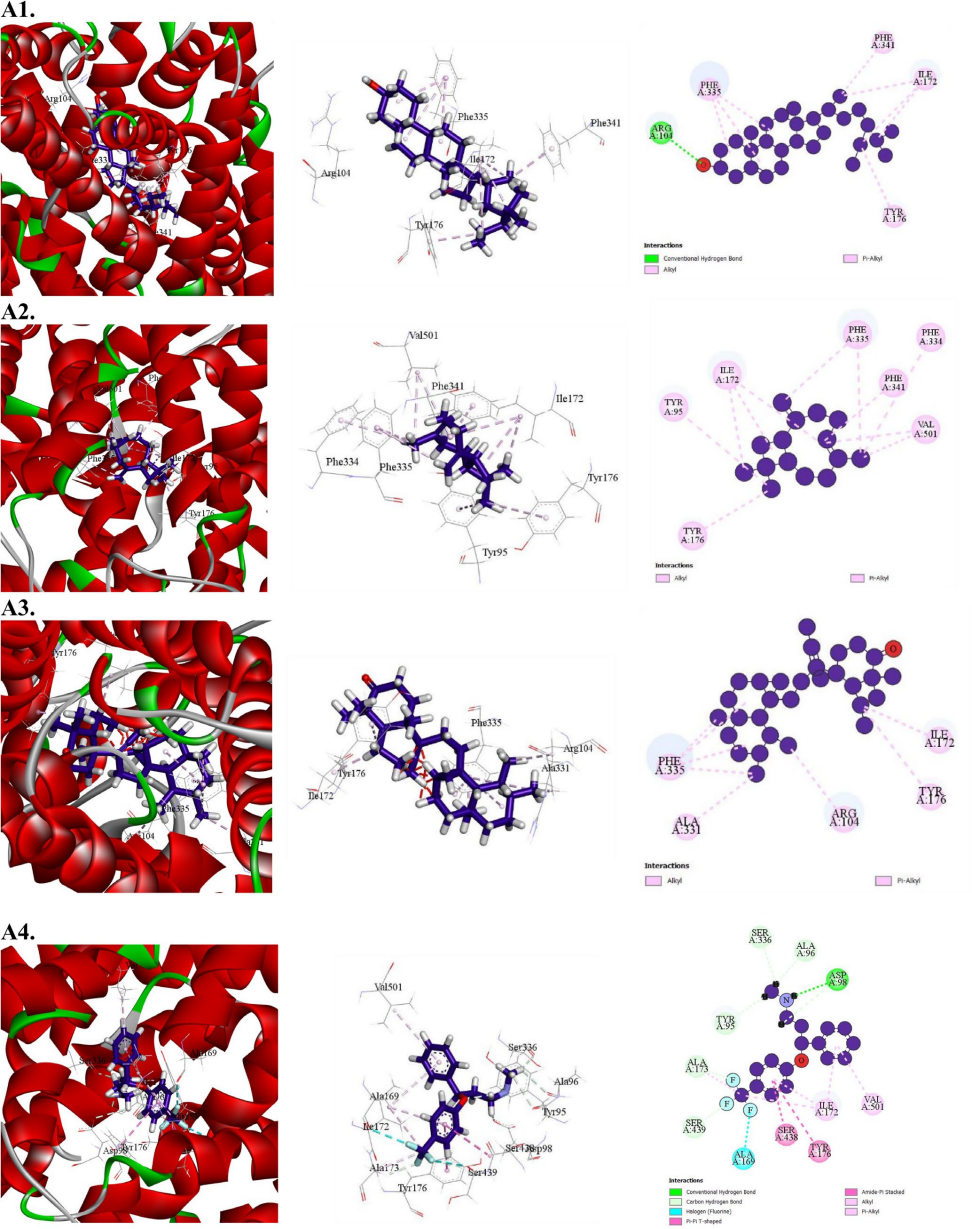
Molecular docking interaction of compounds against the human serotonin transporter (PDB: 5I6X): (A1) gamma‐Sitosterol, (A2) Caryophyllene, (A3) 13,27‐Cycloursan‐3‐one, (A4) Fluoxetine (Standard).

**FIGURE 9:**
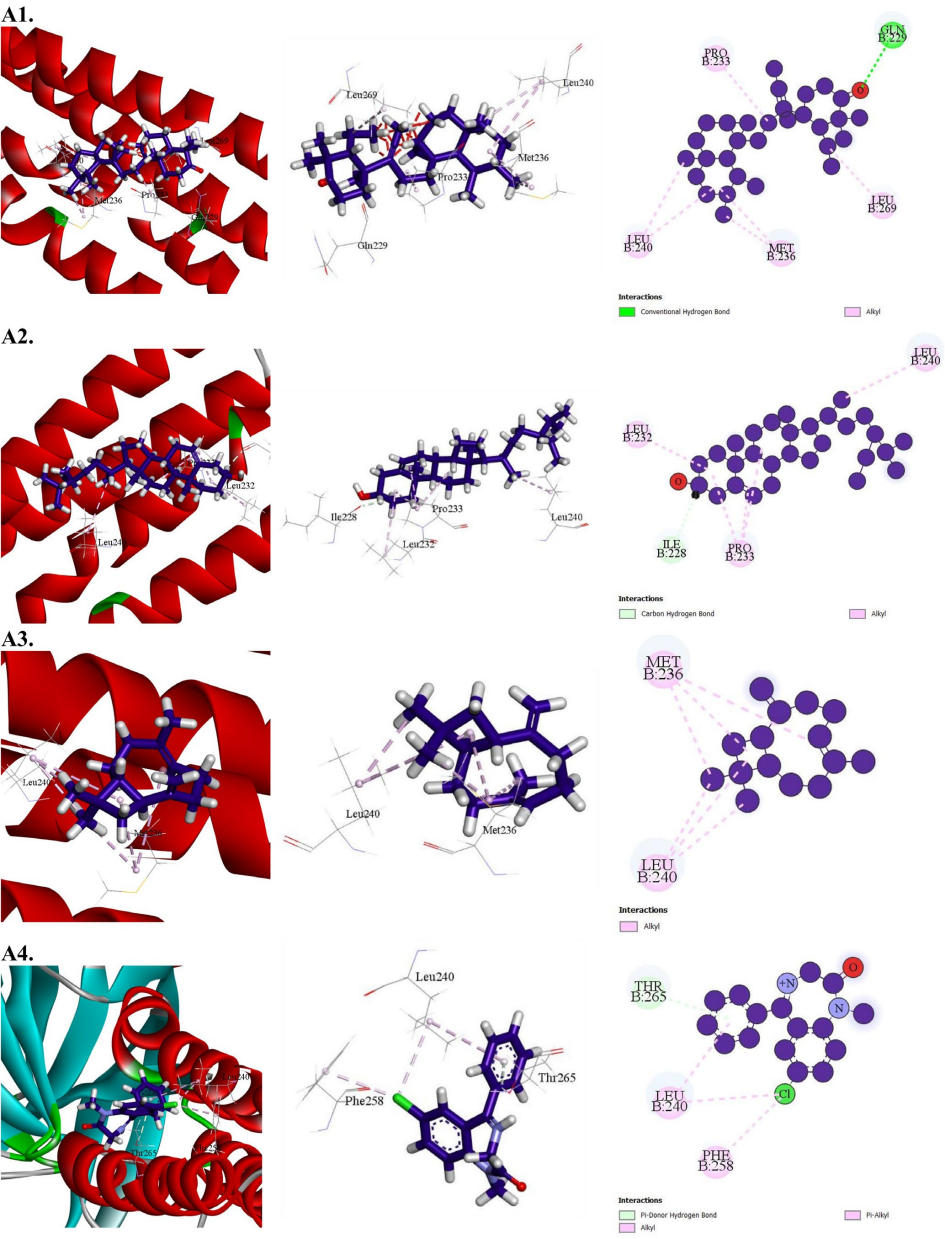
Molecular docking interaction of compounds against the human GABAA receptor alpha1‐beta2‐gamma2 subtype (PDB: 6X3T): (A1) 13,27‐Cycloursan‐3‐one, (A2) γ‐Sitosterol, (A3) Caryophyllene, (A4) Diazepam (Standard).

**FIGURE 10:**
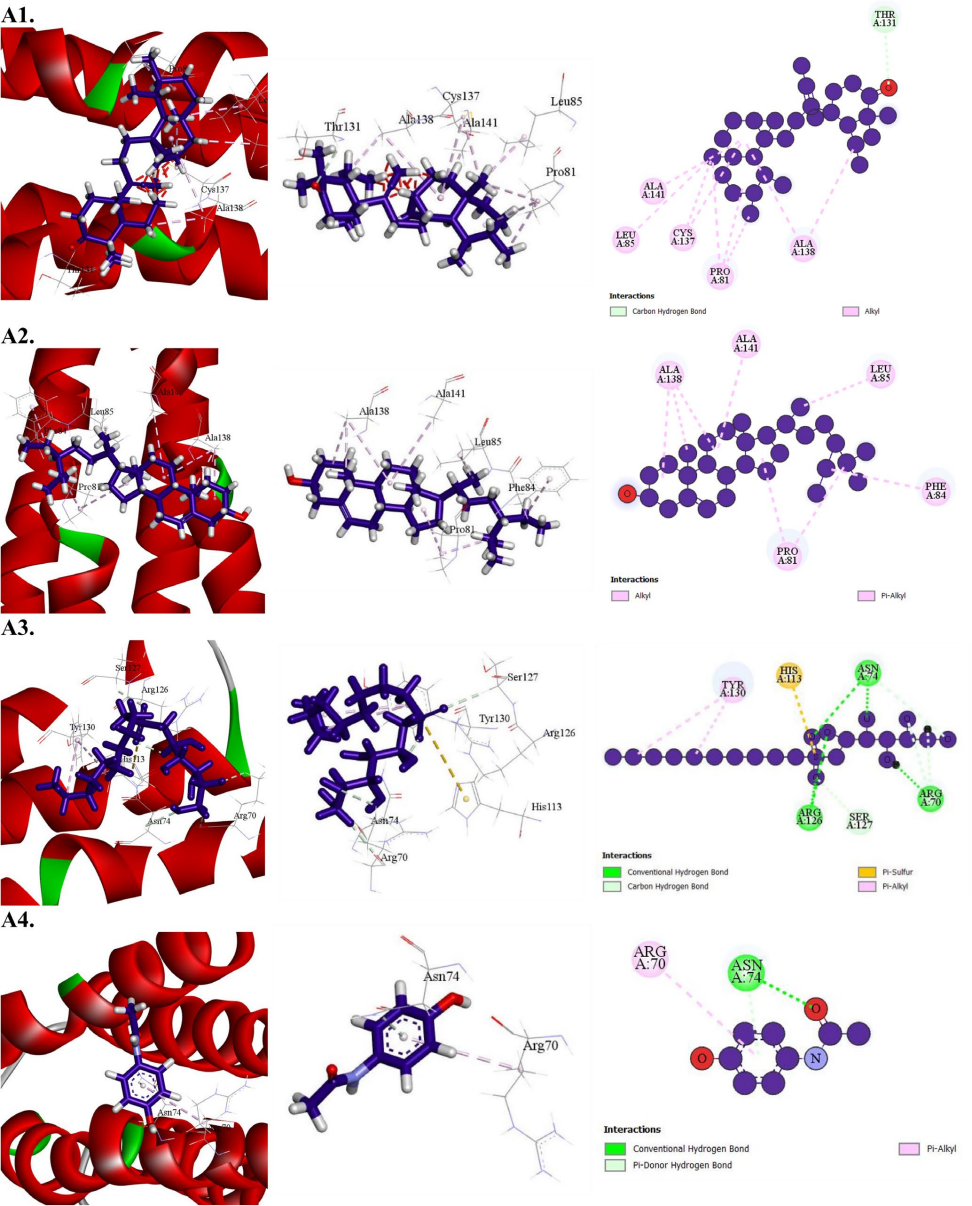
Molecular docking interaction of compounds against the endothiapepsin in complex with fragment 274 (PDB: 4YK5): (A1) 13,27‐Cycloursan‐3‐one, (A2) γ‐Sitosterol, (A3) d‐Mannitol, 1‐decylsulfonyl‐(A4) Paracetamol (Standard).

Molecular docking was employed to investigate the interaction between a specific protein target and the phytochemical components present in MESG. Table 9 summarizes the overall docking scores for each activity. Tables 10, 11, 12, 13 and Figures 7, 8, 9, 10 detail the docking scores and interaction analysis of the top three compounds from MESG for each activity, alongside the reference drug for the corresponding protein targets.

**TABLE 9:** Docking score of the selected compounds identified from the MESG against the human monoamine oxidase (PDB: 2Z5X), human serotonin transporter (PDB: 5I6X), human GABA_A_ receptor alpha1‐beta2‐gamma2 subtype (PDB: 6X3T), and endothiapepsin in complex with fragment 274 (PDB: 4YK5) for anxiolytic, antidepressant, sedative, and antipyretic activity, respectively. MESG, Methanolic Extract of *S. grande* .

Compounds	PubChem CID	Docking score (kcal/mol)			
		Anxiolytic (2z5x)	Antidepressant (5i6x)	Sedative (6x3t)	Antipyretic (4yk5)
2‐O‐Methyl‐d‐xylose	14536300	−4.8	−5	−3.7	−4.1
2‐(Benzylmethylamino)ethanol N‐oxide	264996	−6.6	−6.1	−3.9	−4.4
2‐[2‐[2‐(2‐Butoxyethoxy)ethoxy]ethoxy]ethyl acetate	526863	−6.1	−5.5	−3.7	−3.7
Phytol	5280435	−7.9	−6.9	−4.1	−4.5
9‐Octadecenamide, (Z)—	5283387	−7.7	−6.7	−3.9	−3.6
6,6‐Dimethyl‐cyclohex‐2‐en‐1‐ol	550886	−6.2	−5.2	−3.7	−3.8
Caryophyllene	5281515	−5.8	−8.5	−4.6	−4.6
.gamma.‐Sitosterol	457801	−7.7	**−10**	−5.8	−5.8
Octadecanamide	31292	−7.5	−6.6	−3.5	−3.8
Ethanol, 2‐(octadecyloxy)—	75050	−6.6	−6.4	−3.8	−3.3
1‐Ethyl‐4,4‐dimethyl‐cyclohex‐2‐en‐1‐ol	580366	−6.6	−6.1	−3.9	−4.4
1,2‐Benzenedicarboxylic acid, isodecyl octyl ester	14902	−8	−7.6	−4	−4.3
4′‐Ethoxy‐2′‐hydroxyoctadecanophenone	601846	**−8.5**	−7.3	−3.6	−4.2
Octadecanoic acid, 9,10‐epoxy‐, isopropyl ester	536975	−7.4	−7.4	−3.7	−3.7
d‐Mannitol, 1‐decylsulfonyl—	568528	−7.3	−6.8	−4.4	−4.7
13,27‐Cycloursan‐3‐one	634605	−2.6	−8.3	**−6.9**	**−7.4**
Dodecanamide	14256	−6.4	−6.4	−3.6	−4.1
Cyclopropaneoctanoic acid, 2‐octyl‐, methyl ester	543406	−7.8	−6.7	−3.6	−3.7
Standards (Diazepam, Fluoxetine, Diazepam, Paracetamol)		−8.5	−9.1	−5.1	−4

*Note:* This bold value represents the highest score among the compounds.

**TABLE 10:** Docking scores of the top three compounds identified from the plant, *S. grande,* with the human monoamine oxidase (PDB: 2Z5X).

Compound name	Binding affinity (kcal/mol)	Hydrogen bond interactions				Hydrophobic bond interactions			
		Conventional hydrogen bond		Carbon hydrogen bond		Alkyl		Pi‐Alkyl	
		Amino acid	Distance (Å)	Amino acid	Distance (Å)	Amino acid	Distance (Å)	Amino acid	Distance (Å)
4′‐Ethoxy‐2′‐hydroxyoctadecanophenone	−8.5					ARG51	5.22249	PHE208	4.32666
						CYS323	5.1159	PHE352	5.2831
						CYS406	5.42123	TYR407	4.46034
						ILE335	4.12796	TYR407	5.33239
						LEU337	5.18945	TYR444	5.42475
						ILE335	4.27926		
						LEU337	4.53609		
						LEU97	5.29081		
						CYS323	4.98997		
						ILE325	5.03484		
						ARG51	4.57567		
Octadecanoic acid, 9,10‐epoxy‐, isopropyl ester	−7.4	TYR407	2.69685	GLY67	2.59442	CYS323	5.39895	PHE208	4.13397
		MET445	2.05442	TYR444	2.53243	ILE335	4.30748	PHE352	5.01411
				GLN215	3.03824	ILE337	5.25369	PHE352	5.39208
						ILE335	5.02294	TYR407	4.67427
						ILE337	4.83685	TYR407	4.17884
						CYS323	5.02353		
						ILE325	4.98348		
						MET445	4.85967		
1,2‐Benzenedicarboxylic acid, isodecyl octyl ester	−8	ALA68	2.7514	GLN215	2.85398	ILE180	5.48369	PHE208	4.1809
		TYR69	2.348	TYR69	2.90436	ILE335	4.1577	PHE352	5.3291
						LEU337	5.46317	TYR407	4.85404
						CYS323	5.18232	TYR444	4.85638
						ILE335	4.97736	ALA68	4.93408
						MET445	4.4711		
						ARG51	4.71895		
						MET445	4.93431		
Diazepam (Standard)	−8.5					CYS406	4.38704	TYR407	4.90031
						MET445	4.79125	TYR444	4.26032
								MET445	5.07606

##### 3.7.2.1 Molecular Docking for Anxiolytic Activity

The anxiolytic potential of selected MESG bioactive compounds was evaluated by analyzing their interactions with human monoamine oxidase (PDB: 2Z5X). All compounds demonstrated binding activity with the target receptor. Among them, 4′‐Ethoxy‐2′‐hydroxyoctadecanophenone exhibited the highest binding affinity at −8.5 kcal/mol, followed by Octadecanoic acid, 9,10‐epoxy‐, isopropyl ester (−7.4 kcal/mol), and 1,2‐Benzenedicarboxylic acid, isodecyl octyl ester (−8 kcal/mol). Notably, the binding affinity of 4′‐Ethoxy‐2′‐hydroxyoctadecanophenone matched that of the conventional drug diazepam, which also scored −8.5 kcal/mol. A detailed docking analysis revealed that 4′‐Ethoxy‐2′‐hydroxyoctadecanophenone formed 16 hydrophobic interactions with key amino acid residues, including ARG51 (2), CYS323 (2), CYS406, ILE335 (2), LEU337 (2), LEU97, ILE325, PHE208, PHE352, TYR407 (2), and TYR444. These interactions suggest a strong affinity for the active site of human monoamine oxidase. Shorter bond distances (< 5 Å) were associated with stronger binding and higher docking scores, as hydrophobic contacts primarily drive drug‐receptor interactions (Table 10 and Figure 7).

##### 3.7.2.2 Molecular Docking for Antidepressant Activity

All substances that successfully interacted with the human serotonin transporter (PDB: 5I6X) demonstrated antidepressant efficacy. Among these, gamma‐sitosterol exhibited a binding affinity of −10 kcal/mol, surpassing that of the reference drug fluoxetine, which had a binding affinity of −9.1 kcal/mol (Table 11 and Figure 8). The next highest binding affinities were observed for caryophyllene (−8.5 kcal/mol) and 13,27‐cycloursan‐3‐one (−8.3 kcal/mol). A comprehensive docking analysis revealed that gamma‐sitosterol formed eight hydrophobic interactions with key amino acid residues, including ILE172 (3), TYR176, PHE335 (3), and PHE341, characterized by short intermolecular distances. These findings indicate that gamma‐sitosterol binds strongly to the active site of the human serotonin transporter, highlighting its potential as a potent compound for targeting this receptor.

**TABLE 11:** Docking scores of the top three compounds identified from the plant, *S. grande,* with the human serotonin transporter (PDB ID: 5I6X).

Compound name	Binding affinity (kcal/mol)	Hydrogen bond interactions				Hydrophobic bond interactions			
		Conventional hydrogen bond		Carbon hydrogen bond		Alkyl		Pi‐Alkyl	
		Amino acid	Distance (Å)	Amino Acid	Distance (Å)	Amino acid	Distance (Å)	Amino acid	Distance (Å)
.gamma.‐Sitosterol	−10	ARG104	2.44994			ILE172	4.60259	TYR176	3.84232
						ILE172	4.10846	PHE335	5.30777
						ILE172	5.23413	PHE335	5.24347
								PHE335	4.12283
								PHE341	3.69395
Caryophyllene	−8.5					ILE172	4.57808	TYR95	5.20531
						ILE172	4.87749	TYR176	5.10088
						VAL501	4.78465	PHE334	5.10088
						ILE172	4.26459	PHE335	5.48407
						VAL501	4.70308	PHE335	4.74144
								PHE341	4.91439
13,27‐Cycloursan‐3‐one	−8.3					ALA331	4.31467	TYR176	4.71021
						ILE172	3.49301	PHE335	4.81482
						ARG104	4.41797	PHE335	4.9494
								PHE335	3.36475
								PHE335	5.31548
Fluoxetine (Standard)	−9.1	ASP98	2.70102	ALA173	2.52772	ALA173	4.06346	ILE172	4.61225
				SER439	2.50192	ILE172	4.90709	ILE172	5.2852
				ASP98	3.09673			VAL501	5.23446
				TYR95	3.03392				
				ALA96	2.89288				
				SER336	2.42107				

##### 3.7.2.3 Molecular Docking for Sedative Activity

In the context of sedative activity, all compounds demonstrated affinity for the human GABA_A_ receptor alpha1–beta2–gamma2 subtype (PDB: 6X3T). Among them, 13,27‐Cycloursan‐3‐one exhibited the highest binding affinity at −6.9 kcal/mol, surpassing the reference drug diazepam, which had a binding affinity of −5.1 kcal/mol (Table 12 and Figure 9). The next strongest binders were gamma‐Sitosterol (−5.8 kcal/mol) and Caryophyllene (−4.6 kcal/mol). A detailed docking analysis revealed that 13,27‐Cycloursan‐3‐one formed six hydrophobic interactions with key amino acid residues, including PRO233, MET236 (3), LEU269, and LEU240, characterized by short intermolecular distances. These findings indicate that 13,27‐Cycloursan‐3‐one exhibits a strong affinity for the active site of the human GABA_A_ receptor alpha1–beta2–gamma2 subtype, suggesting its potential as a promising compound for targeting this receptor.

**TABLE 12:** Docking scores of the top three compounds identified from the plant, *S. grande,* with the human GABA_A_ receptor alpha1‐beta2‐gamma2 subtype (PDB: 6X3T).

Compound name	Binding affinity (kcal/mol)	Hydrogen bond interactions				Hydrophobic bond interactions			
		Conventional hydrogen bond		Carbon hydrogen bond		Alkyl		Pi‐Alkyl	
		Amino acid	Distance (Å)	Amino acid	Distance (Å)	Amino acid	Distance (Å)	Amino acid	Distance (Å)
13,27‐Cycloursan‐3‐one	−6.9	GLN229	2.99446			PRO233	4.26573		
						MET236	5.26186		
						MET236	5.4212		
						LEU269	5.32954		
						LEU240	4.9081		
						MET236	3.75833		
.gamma.‐Sitosterol	−5.8			ILE228	2.1992	PRO233	4.53183		
						PRO233	4.02756		
						PRO233	4.46949		
						LEU232	4.92114		
						LEU240	4.95723		
Caryophyllene	−4.6					MET236	4.84905		
						MET236	5.30831		
						LEU240	5.06441		
						LEU240	3.89295		
						MET236	4.12743		
						LEU240	4.00862		
Diazepam (Standard)	−5.1					LEU240	4.20698	PHE258	3.86323
								LEU240	4.80945

##### 3.7.2.4 Molecular Docking for Antipyretic Activity

All compounds demonstrated effective interactions with the target receptor, microsomal prostaglandin E synthase 1 (mPGES‐1) (PDB: 4YK5). The highest binding affinity was observed for 13,27‐Cycloursan‐3‐one, with a score of −7.4 kcal/mol. The next strongest binders were gamma‐Sitosterol (−5.8 kcal/mol) and d‐Mannitol, 1‐decylsulfonyl (−4.7 kcal/mol). A detailed docking analysis revealed that 13,27‐Cycloursan‐3‐one formed nine hydrophobic bonds with key amino acid residues, including PRO81 (3), CYS137 (2), ALA138 (2), ALA141, and LEU85, characterized by short intermolecular distances. These findings indicate that 13,27‐Cycloursan‐3‐one exhibits a strong affinity for microsomal prostaglandin E synthase 1 (mPGES‐1), highlighting its potential as a highly effective compound for targeting this receptor (Table 13 and Figure 10).

**TABLE 13:** Docking scores of the top three compounds identified from the plant, *S. grande,* with the human serotonin transporter (PDB ID: 4YK5).

Compound name	Binding affinity (kcal/mol)	Hydrogen bond interactions				Hydrophobic bond interactions			
		Conventional hydrogen bond		Carbon hydrogen bond		Alkyl		Pi‐Alkyl	
		Amino acid	Distance (Å)	Amino acid	Distance (Å)	Amino Acid	Distance (Å)	Amino Acid	Distance (Å)
13,27‐Cycloursan‐3‐one	−7.4			THR131	2.91708	PRO81	4.92852		
						CYS137	5.48895		
						ALA138	5.44002		
						ALA138	4.03817		
						ALA141	5.42981		
						PRO81	4.45016		
						LEU85	4.59758		
						CYS137	4.32085		
						PRO81	4.28616		
.gamma.‐Sitosterol	−5.8					PRO81	5.06954	PHE84	4.23263
						ALA138	5.21043		
						ALA138	4.70953		
						ALA138	3.75279		
						ALA141	5.31627		
						LEU85	4.84092		
						PRO81	4.13999		
d‐Mannitol, 1‐decylsulfonyl‐	−4.7	ASN74	2.64287	ARG70	2.7222			TYR130	5.05842
		ASN74	2.01389	ASN74	2.98739			TYR130	5.35353
		ARG126	2.92221	SER127	2.78794				
		ARG126	2.60347	ARG70	2.51419				
		ARG126	2.91727						
		ARG126	2.17747						
		ARG126	2.62186						
Paracetamol (Standard)	−4	ASN74	2.66656					ARG70	5.40128

### 3.8 Pass Prediction

The anxiolytic, depressive, sedative, and antipyretic properties of 18 carefully selected MESG compounds were investigated using the PASS online tool. The findings showed that substances with significant molecular potency had Pa values higher than Pi (Table 14).

**TABLE 14:** Biological Activity of MESG through Pass Prediction Analysis. MESG = Methanolic Extract of *S. grande* .

Compounds	Biological activity							
	Anxiolytic		Antidepressant		Sedative		Antipyretic	
	Pa	Pi	Pa	Pi	Pa	Pi	Pa	Pi
2‐O‐Methyl‐d‐xylose	—	—	—	—	0.222	0.065	0.203	0.111
2‐(Benzylmethylamino)ethanol N‐oxide	0.295	0.065	**0.347**	0.013	0.392	0.022	—	—
2‐[2‐[2‐(2‐Butoxyethoxy)ethoxy]ethoxy]ethyl acetate	0.326	0.047	0.157	0.084	0.161	0.071	0.532	0.010
Phytol	0.210	0.136	—	—	0.241	0.057	—	—
9‐Octadecenamide, (Z)—	0.205	0.141	—	—	0.501	0.011	0.231	0.087
6,6‐Dimethyl‐cyclohex‐2‐en‐1‐ol	0.230	0.115	—	—	0.404	0.020	—	—
Caryophyllene	—	—	—	—	0.138	0.129	—	—
.gamma.‐Sitosterol	—	—	—	—	—	—	—	—
Octadecanamide	0.289	0.069	—	—	**0.535**	0.009	0.248	0.073
Ethanol, 2‐(octadecyloxy)—	0.333	0.044	0.178	0.072	0.147	0.088	0.418	0.024
1‐Ethyl‐4,4‐dimethyl‐cyclohex‐2‐en‐1‐ol	0.296	0.065	—	—	0.180	0.053	—	—
1,2‐Benzenedicarboxylic acid, isodecyl octyl ester	**0.403**	0.021	—	—	0.147	0.117	0.328	0.037
4′‐Ethoxy‐2′‐hydroxyoctadecanophenone	0.079	0.034	—	—	0.142	0.124	**0.790**	0.004
Octadecanoic acid, 9,10‐epoxy‐, isopropyl ester	—	—	—	—	0.160	0.105	0.184	0.131
d‐Mannitol, 1‐decylsulfonyl—	—	—	—	—	0.215	0.046	—	—
13,27‐Cycloursan‐3‐one	—	—	—	—	—	—	—	—
Dodecanamide	0.289	0.069	—	—	**0.535**	0.009	0.248	0.073
Cyclopropaneoctanoic acid, 2‐octyl‐, methyl ester	0.225	0.120	0.161	0.081	0.164	0.102	0.259	0.065

*Note:* This bold value represents the highest score among the compounds.

## 4 Discussion

Medicinal plants have been a source of remedies for various health conditions for centuries. Their use dates back to ancient times, where they were extensively relied upon as safe and effective treatments for human ailments, with their applications well recorded throughout history (Vonshak et al. 2003). Globally, research into medicinal plants has made consistent progress, driven by the search for new therapeutic options to address neurological disorders. This research has demonstrated the pharmacological potential of numerous plant species through testing in various animal models (Zhang 2004). In this particular study, we assessed the effects of MESG on anxiolytic, antidepressant, sedative, and antipyretic activities in animals. Additionally, we identified bioactive compounds in the extract using GC–MS/MS analysis.

For assessing anxiety‐related behavior, one of the most popular animal models is the Elevated Plus Maze (EPM) test. The idea behind it is that mice avoid open and high regions of the maze and prefer enclosed (closed arm) areas, as they drive them to feel frightened and anxious. Exploratory behavior is usually encouraged by anxiolytic treatment, which results in more time spent and more entries into the maze's open arms (Walf and Frye 2007). Diazepam (1 mg/kg) and the extract MESG at 200 and 400 mg/kg showed notable anxiolytic effects in this investigation by lengthening the duration and increasing the number of admissions into the open arms. An alternate technique for evaluating anxiolytic activity is the hole‐board test. This test uses a specific hole‐board setup to measure the animals' head‐dipping habit. Reduced anxiety or emotional state changes are linked to increased head‐dipping behavior (Eva et al. 2024). In contrast to the control group (19.2 ± 0.86), MESG at a dose of 400 mg/kg considerably (*p* < 0.001) increased the number of head‐dips (41.2 ± 1.46). The anxiolytic effects of MESG were also examined using the Light–Dark Box (LDB) test. A bright (aversive) and a dark (safe) container comprise the LDB device. Despite their innate aversion to brightly lit environments, mice can spend more time in the light compartment and less time in the dark compartment when taking anxiolytic medications (Bourin and Hascoët 2003). At doses of 200 and 400 mg/kg, MESG markedly reduced the amount of time spent in the dark box and increased the amount of time spent in the light box. At 400 mg/kg, the effects of MESG were similar to those of diazepam. When compared to the control group, MESG at 400 mg/kg demonstrated noteworthy anxiolytic efficacy in all three tests. Alkaloids, tannins, phenols, and flavonoids were among the structurally diverse secondary metabolites identified in *S. grande* leaves (MESG) through GC–MS/MS analysis. Alkaloids, flavonoids, and phenols have been shown in earlier research to have anxiolytic effects because of their high affinity for the GABA_A_ receptor's benzodiazepine (BZD)‐binding region (Fedotova et al. 2017). In the central nervous system (CNS), gamma‐aminobutyric acid (GABA) acts as a primary inhibitory neurotransmitter. Benzodiazepines exert their effects by binding to GABAA receptors, which enhances the receptor's response to GABA. This facilitates an increased influx of chloride ions into neurons, resulting in hyperpolarization of the neuronal membrane and a reduction in neuronal excitability. This neurophysiological mechanism underlies the anxiolytic effects of benzodiazepines (Haefely 1984). The bioactive compounds identified in MESG have the potential to interact with the BZD‐binding site of GABA_A_ receptors, thereby producing anxiolytic effects.

The antidepressant effects of MESG were assessed in mice using two well‐established behavioral models: the Tail Suspension Test (TST) and the Forced Swimming Test (FST). In these tests, when animals are placed in stressful, inescapable situations, they tend to exhibit immobility for extended periods. This immobility is interpreted as a sign of despair, hopelessness, or an inability to adapt to the stressful environment, which mirrors depressive‐like behavior (Can et al. 2012) (Yankelevitch‐Yahav et al. 2015). Treatment with antidepressants typically reduces the duration of immobility (Shah et al. 2022). In this study, MESG demonstrated mild antidepressant activity by reducing immobility time compared to untreated animals in the FST model. At a dose of 400 mg/kg, MESG produced a moderate but significant (*p* < 0.01) reduction in immobility, although its effect was less pronounced than that of the standard antidepressant fluoxetine in both the TST and FST. Several mechanisms have been proposed to explain the pathophysiology of depression, with a central focus on the dysregulation of key neurotransmitter systems in the central nervous system (CNS), particularly serotonin and norepinephrine. This imbalance is thought to contribute to the mood and cognitive symptoms associated with depression. Various classes of antidepressants target these pathways to restore normal neurotransmission. Selective serotonin reuptake inhibitors (SSRIs) and serotonin‐norepinephrine reuptake inhibitors (SNRIs) exert their effects by blocking the reuptake of serotonin and, in the case of SNRIs, norepinephrine as well, thereby increasing their availability in the synaptic cleft. Meanwhile, monoamine oxidase inhibitors (MAOIs) prevent the breakdown of these neurotransmitters by inhibiting the activity of monoamine oxidase enzymes, further enhancing their levels and signaling in the brain (Shah et al. 2022). Additionally, depression has been linked to excessive activation of the hypothalamic–pituitary–adrenal (HPA) axis, which triggers the release of stress‐related neuropeptides like corticotropin‐releasing factor (CRF) (Jiang et al. 2019). The compounds present in MESG may exert antidepressant effects by modulating these pathways, potentially inhibiting neurotransmitter reuptake, acting as MAO inhibitors, or mitigating the effects of HPA axis overactivation. These mechanisms could contribute to the observed antidepressant activity of the extract.

The sedative effects of MESG were assessed using two widely used animal models: the Open Field Test (OFT) and the Hole Cross Test (HCT), which measure unrestrained locomotor activity. Diazepam, a benzodiazepine and central nervous system (CNS) depressant, is commonly prescribed for insomnia and other sleep disorders. It acts on the GABA receptor complex, specifically at the benzodiazepine‐binding site, to reduce activity, control excitability, and induce relaxation. Benzodiazepines also decrease exploratory behavior and prolong barbiturate‐induced sleep (M. F. Hossain et al. 2016). The findings of this study revealed that MESG produced notable behavioral changes indicative of sedative effects in both tests. Specifically, there was a notable reduction in the frequency of movements between chambers and hole crossings, reflecting decreased locomotor activity in mice. Compared to the control group, MESG administered at doses of 200 and 400 mg/kg resulted in a dose‐dependent reduction in square movements in the OFT. Similarly, in the HCT, the number of hole crossings and square movements decreased significantly between 30 and 120 min postadministration. These behavioral alterations suggest that MESG may contain bioactive compounds capable of modulating motor function and reducing spontaneous activity in mice. The observed dose‐dependent reduction in locomotion indicates that MESG likely exerts sedative and CNS‐depressant effects by enhancing GABAergic inhibition, potentially through increased hyperpolarization of neuronal membranes. This could involve activation of GABA receptors or suppression of neuronal activity (Gahlot et al. 2013). However, the specific components responsible for these effects remain unclear, as further research is needed to identify and evaluate the roles of individual compounds isolated from the extract in producing the observed sedative activity.

The subcutaneous injection of Brewer's yeast induces fever by increasing prostaglandin production, making it a promising model for screening both natural and synthetic antipyretic agents. This type of pyrexia, triggered by fungal exposure, is considered pathogenic, with prostaglandins playing a key role in its development (Saper and Breder 1994). The antipyretic effect may involve the inhibition of cyclooxygenase enzyme activity, which blocks prostaglandin synthesis—a mechanism similar to that of paracetamol. Effective suppression of pyrexia‐related mediators in the body appears to be the primary factor responsible for antipyretic action, rather than any single specific element (Zishan et al. 2023). In this study, intraperitoneal administration of MESG significantly lowered the rectal temperature in yeast‐induced febrile mice. These results suggest that the extract contains pharmacologically active compounds that may exert their effect by inhibiting prostaglandin synthesis or release, a key pathway in fever development. The observed antipyretic effect demonstrates the therapeutic potential of MESG in managing fever. Overall, the findings indicate that MESG possesses bioactive constituents with significant antipyretic properties, as confirmed by the marked reduction in body temperature in the treated animal model.

Globally, biological research is acknowledged as a very promising topic, and biomedical research is increasingly dependent on sophisticated informatics technologies like database mining and high‐throughput screening. Multiple bioinformatics tools have recently been developed to help a range of biological research topics, such as secretome analysis, cancer metabolomics, and epitope modeling (Hossen et al. 2022). Among these tools, molecular docking has emerged as a critical technique in structural biology. Molecular docking analyzes the interaction between selected ligand molecules (compounds of interest) and specific protein receptors (Morris and Lim‐Wilby 2008). This study employed molecular docking to evaluate the binding affinity of selected compounds isolated from MESG via GC–MS/MS analysis. These compounds were docked with key protein targets associated with anxiolytic, antidepressant, sedative, and antipyretic activities: human monoamine oxidase (PDB: 2Z5X), human serotonin transporter (PDB: 5I6X), human GABA_A_ receptor alpha1‐beta2‐gamma2 subtype (PDB: 6X3T), and microsomal prostaglandin E synthase 1 (mPGES‐1) (PDB: 4YK5). The results revealed that 4′‐Ethoxy‐2′‐hydroxyoctadecanophenone (−8.5 kcal/mol), γ‐Sitosterol (−10 kcal/mol), and 13,27‐Cycloursan‐3‐one (−6.9 and −7.4 kcal/mol) exhibited the highest docking scores, respectively, with their corresponding protein targets. These scores suggest that these compounds likely interact more effectively with the target receptors than other bioactive constituents identified in the extract. However, further experimental validation is necessary to confirm the pharmacological potential and therapeutic implications of these isolated compounds.

The results suggest that the phytochemicals meet Lipinski's Rule of Five, indicating their potential suitability for oral administration and highlighting their promise as therapeutic candidates. Since drug safety is a critical aspect of developing high‐quality medicinal products, we further evaluated the toxicological profiles of the identified plant compounds using the admetSAR online tool. This assessment aimed to ensure the safety and reliability of these compounds for potential pharmaceutical applications (Cheng et al. 2012).

A computational approach known as PASS was employed to elucidate the diverse biological effects of the compounds by simulating their activities based on varying levels of bioactivity (Poroikov and Filimonov 2005). In this study, a compound was considered suitable for a specific biological activity if its Pa value (probability of trustworthy activity) exceeded its Pi value (probability of trustworthy inactivity). This rigorous analysis yielded significant insights into the selected compounds. Notably, 4′‐Ethoxy‐2′‐hydroxyoctadecanophenone demonstrated high potential as an antipyretic agent, with a maximal Pa value of 0.790. The observed outcomes may be attributed to the combined action of multiple phytochemicals, including both previously reported and unreported compounds, which could contribute synergistically to the overall biological effects.

The limited availability of resources and the restricted scope of LC–MS (liquid chromatography–mass spectrometry) were notable limitations of this study. These constraints hindered the in‐depth analysis and identification of certain compounds that could have offered more profound insights into the phytochemical profile and bioactive potential of the extract. Nevertheless, alternative tools such as gas chromatography–mass spectrometry (GC–MS/MS) were utilized to the best of their capacity to explore the chemical composition and associated biological activities of the extract. Future studies with access to LC–MS are recommended to overcome these limitations and achieve a more comprehensive characterization of the extract, potentially uncovering additional bioactive components and enhancing the understanding of its therapeutic properties.

## 5 Conclusion

The current study highlights the significant sedative, antipyretic, and anxiolytic properties of the methanolic extract of *S. grande* leaves (MESG), along with moderate antidepressant effects. GC–MS/MS analysis identified 64 compounds in MESG, including bioactive molecules such as phytol, which are known for their neuropharmacological activities. In silico studies further demonstrated that these plant‐derived compounds exhibit biological activity, interact with key enzymes in human physiological systems, and possess drug‐like characteristics.

However, the pharmacological effects observed may also be attributed to unidentified compounds within the extract. To better understand the precise mechanisms underlying these effects, further research is recommended to investigate the phytoconstituents of *S. grande* in greater detail. Such studies could pave the way for the development of novel therapeutic agents derived from this plant.

## Author Contributions

**Md. Jahirul Islam Mamun:** conceptualization (equal), formal analysis (equal), investigation (equal), methodology (equal), project administration (equal), software (equal), visualization (equal), writing – original draft (equal), writing – review and editing (equal). **Sifatul Islam Mizan:** data curation (equal), formal analysis (equal), investigation (equal), methodology (equal), software (equal), writing – original draft (equal), writing – review and editing (equal). **Mahathir Mohammad:** data curation (equal), formal analysis (equal), investigation (equal), resources (equal), software (equal), writing – original draft (equal). **Md. Liakot Ali:** data curation (equal), formal analysis (equal), investigation (equal), resources (equal), software (equal), writing – original draft (equal). **Md. Tanveer Ahsan:** conceptualization (equal), project administration (equal), resources (equal), supervision (equal), validation (equal).

## Conflicts of Interest

The authors declare no conflicts of interest.

## Data Availability

The data supporting the study's findings will be made available by the corresponding author upon reasonable request.

## References

[fsn371088-bib-0001] Abdusalam, K. B. , L. S. Yee , A. Mediani , et al. 2022. “1H NMR‐Based Metabolomics Profiling of *Syzygium grande* and *Oenanthe javanica* and Relationship Between Their Metabolite Compositions and Antimicrobial Activity Against Bacillus Species.” Records of Natural Products 16: 128–143.

[fsn371088-bib-0002] Adnan, M. , M. Nazim Uddin Chy , A. T. M. Mostafa Kamal , et al. 2019. “Investigation of the Biological Activities and Characterization of Bioactive Constituents of Ophiorrhiza Rugosa Var. Prostrata (D. Don) & Mondal Leaves Through In Vivo, In Vitro, and In Silico Approaches.” Molecules 24, no. 7: 1367. 30965575 10.3390/molecules24071367 PMC6480688

[fsn371088-bib-0003] Ali, M. L. , J. N. Meem , N. Hoque , et al. 2025. “GC–MS Analysis, Neuropharmacological and Antidiarrheal Activities of the Acetone Extract of *Najas gracillima* Seaweed: In Vivo and In Silico Study.” Chemistry & Biodiversity 22: e202402303. 39714997 10.1002/cbdv.202402303

[fsn371088-bib-0004] Ali, M. L. , F. Noushin , E. Azme , M. M. Hasan , N. Hoque , and A. F. Metu . 2024. “Marine Natural Compounds as Potential CBP Bromodomain Inhibitors for Treating Cancer: An In Silico Approach Using Molecular Docking, ADMET, Molecular Dynamics Simulations and MM‐PBSA Binding Free Energy Calculations.” In Silico Pharmacology 12, no. 2: 1–20. 39310674 10.1007/s40203-024-00258-5 PMC11411048

[fsn371088-bib-0005] Aronoff, D. M. , and E. G. Neilson . 2001. “Antipyretics: Mechanisms of Action and Clinical Use in Fever Suppression.” American Journal of Medicine 111, no. 4: 304–315. 11566461 10.1016/s0002-9343(01)00834-8

[fsn371088-bib-0006] Ayoola, G. A. , F. M. Lawore , T. Adelowotan , et al. 2008. “Chemical Analysis and Antimicrobial Activity of the Essential Oil of Syzigium Aromaticum (Clove).” African Journal of Microbiology Research 2, no. 7: 162–166.

[fsn371088-bib-0007] Azme, E. , M. M. Hasan , M. L. Ali , et al. 2025. “Computational Identification of Potential Natural Terpenoid Inhibitors of MDM2 for Breast Cancer Therapy: Molecular Docking, Molecular Dynamics Simulation, and ADMET Analysis.” Frontiers in Chemistry 13: 1527008. 40308267 10.3389/fchem.2025.1527008 PMC12041027

[fsn371088-bib-0008] Bourin, M. , and M. Hascoët . 2003. “The Mouse Light/Dark Box Test.” European Journal of Pharmacology 463, no. 1–3: 55–65. 12600702 10.1016/s0014-2999(03)01274-3

[fsn371088-bib-0009] Cai, L. , and C. D. Wu . 1996. “Compounds From *Syzygium aromaticum* Possessing Growth Inhibitory Activity Against Oral Pathogens.” Journal of Natural Products 59, no. 10: 987–990. 8904847 10.1021/np960451q

[fsn371088-bib-0010] Can, A. , D. T. Dao , C. E. Terrillion , S. C. Piantadosi , S. Bhat , and T. D. Gould . 2012. “The Tail Suspension Test.” Journal of Visualized Experiments 59: e3769. 10.3791/3769 PMC3353516 22315011

[fsn371088-bib-0011] Chaudhuri, A. K. N. , S. Pal , A. Gomes , and S. Bhattacharya . 1990. “Anti‐Inflammatory and Related Actions of *Syzygium cuminii* Seed Extract.” Phytotherapy Research 4, no. 1: 5–10.

[fsn371088-bib-0012] Cheng, F. , W. Li , Y. Zhou , et al. 2012. AdmetSAR: A Comprehensive Source and Free Tool for Assessment of Chemical ADMET Properties. Acs Publications. 10.1021/ci300367a 23092397

[fsn371088-bib-0013] Daina, A. , O. Michielin , and V. Zoete . 2017. “SwissADME: A Free Web Tool to Evaluate Pharmacokinetics, Drug‐Likeness and Medicinal Chemistry Friendliness of Small Molecules.” Scientific Reports 7, no. 1: 42,717. 28256516 10.1038/srep42717 PMC5335600

[fsn371088-bib-0014] de Oliveira, G. F. , N. A. J. C. Furtado , A. A. da Silva Filho , et al. 2007. “Antimicrobial Activity of *Syzygium cumini* (Myrtaceae) Leaves Extract.” Brazilian Journal of Microbiology 38: 381–384.

[fsn371088-bib-0015] Dhawan, K. , S. Dhawan , and S. Chhabra . 2003. “Attenuation of Benzodiazepine Dependence in Mice by a Tri‐Substituted Benzoflavone Moiety of *Passiflora incarnata* Linneaus: A Non‐Habit Forming Anxiolytic.” Journal of Pharmacy & Pharmaceutical Sciences 6, no. 2: 215–222. 12935433

[fsn371088-bib-0016] Dhingra, D. , and A. Sharma . 2006. “A Review on Antidepressant Plants.” https://nopr.niscpr.res.in/bitstream/123456789/7987/1/NPR%205(2)%20144‐152.pdf.

[fsn371088-bib-0018] Emon, N. U. , S. Alam , S. Rudra , et al. 2021. “Antidepressant, Anxiolytic, Antipyretic, and Thrombolytic Profiling of Methanol Extract of the Aerial Part of *Piper nigrum* : In Vivo, In Vitro, and In Silico Approaches.” Food Science & Nutrition 9, no. 2: 833–846. 33598167 10.1002/fsn3.2047 PMC7866625

[fsn371088-bib-0017] Emon, N. U. , S. Alam , S. Rudra , S. Chowdhury , J. C. Rajbangshi , and A. Ganguly . 2022. “Evaluation of Pharmacological Potentials of the Aerial Part of *Achyranthes aspera* L.: In Vivo, In Vitro and In Silico Approaches.” Advances in Traditional Medicine 22, no. 1: 141–154.

[fsn371088-bib-0019] Emon, N. U. , M. Kaiser , M. Islam , et al. 2020. “Anxiolytic and Thrombolytic Investigation of Methanol Extract of *Piper nigrum* L. Fruits and *Sesamum indicum* L. Seeds.” Journal of Advanced Biotechnology and Experimental Therapeutics 3, no. 3: 158–164.

[fsn371088-bib-0020] Estella, O. U. , A. C. William , O. Patrick , et al. 2022. “Evaluation of the Analgesic and Antipyretic Activity of Methanol Extract of Combretum Bauchiense Hutch & Dalziel (Combretaceae) Leaves.” Phytomedicine Plus 2, no. 1: 100–166.

[fsn371088-bib-0021] Eva, T. A. , H. Mamurat , M. H. H. Rahat , and S. M. M. Hossen . 2024. “Unveiling the Pharmacological Potential of Coelogyne Suaveolens: An Investigation of Its Diverse Pharmacological Activities by In Vivo and Computational Studies.” Food Science & Nutrition 12, no. 3: 1749–1767. 10.1002/fsn3.3867. 38455216 PMC10916579

[fsn371088-bib-0022] Fedotova, J. , P. Kubatka , D. Büsselberg , et al. 2017. “Therapeutical Strategies for Anxiety and Anxiety‐Like Disorders Using Plant‐Derived Natural Compounds and Plant Extracts.” Biomedicine & Pharmacotherapy 95: 437–446. 28863384 10.1016/j.biopha.2017.08.107

[fsn371088-bib-0023] Gahlot, K. , V. K. Lal , and S. Jha . 2013. “Anticonvulsant Potential of Ethanol Extracts and Their Solvent Partitioned Fractions From *Flemingia strobilifera* Root.” Pharmacognosy Research 5, no. 4: 265–270. 24174820 10.4103/0974-8490.118825 PMC3807991

[fsn371088-bib-0024] Guedes, I. A. , C. S. de Magalhães , and L. E. Dardenne . 2014. “Receptor–Ligand Molecular Docking.” Biophysical Reviews 6: 75–87. 28509958 10.1007/s12551-013-0130-2 PMC5425711

[fsn371088-bib-0025] Gupta, B. D. , P. C. Dandiya , and M. L. Gupta . 1971. “A Psycho‐Pharmacological Analysis of Behaviour in Rats.” Japanese Journal of Pharmacology 21, no. 3: 293–298. 4397666 10.1254/jjp.21.293

[fsn371088-bib-0026] Haefely, W. 1984. “Benzodiazepine Interactions With GABA Receptors.” Neuroscience Letters 47, no. 3: 201–206. 6147796 10.1016/0304-3940(84)90514-7

[fsn371088-bib-0027] Hasan, M. M. , E. Azme , R. Alam , et al. 2025. “Mushrooms as Potent Autophagy Modulators in Cancer Therapy: Current Evidence and Therapeutic Prospects.” Cancer Pathogenesis and Therapy 3: E01–E58. 10.1016/j.cpt.2025.08.001. PMC12992099 41853122

[fsn371088-bib-0028] Hossain, M. F. , B. Talukder , M. N. Rana , et al. 2016. “In Vivo Sedative Activity of Methanolic Extract of Stericulia Villosa Roxb. Leaves.” BMC Complementary and Alternative Medicine 16: 1–4. 27769218 10.1186/s12906-016-1374-8 PMC5073963

[fsn371088-bib-0029] Hossain, S. , S. A. H. Rabbi , M. J. I. Mamun , et al. 2025. “Antioxidant, Anti‐Inflammatory, and Neuropharmacological Potential of *Syngonium podophyllum* Flower Methanolic Extract: Insights From In Vivo, In Vitro, In Silico, and GC–MS/MS Analysis.” Chemistry & Biodiversity 22: e202500425. 10.1002/cbdv.202500425. 40207503

[fsn371088-bib-0030] Hossen, S. M. M. , M. S. Hossain , S. Akbar , U. Tahmida , J. Mawa , and N. U. Emon . 2021. “Wild Mushrooms Showed Analgesic and Cytotoxic Properties Along With Phytoconstituent's Binding Affinity to COX‐1, COX‐2 and Cytochrome P450 2C9.” Heliyon 7, no. 9: e07997. 34585013 10.1016/j.heliyon.2021.e07997 PMC8455681

[fsn371088-bib-0031] Hossen, S. M. M. , M. S. Hossain , A. T. M. Yusuf , P. Chaudhary , N. U. Emon , and P. Janmeda . 2022. “Profiling of Phytochemical and Antioxidant Activity of Wild Mushrooms: Evidence From the In Vitro Study and Phytoconstituent's Binding Affinity to the Human Erythrocyte Catalase and Human Glutathione Reductase.” Food Science & Nutrition 10, no. 1: 88–102. 35035912 10.1002/fsn3.2650 PMC8751451

[fsn371088-bib-0032] Hossen, S. M. M. , M. J. Islam , M. R. Hossain , A. Barua , M. G. Uddin , and N. U. Emon . 2021. “CNS Anti‐Depressant, Anxiolytic and Analgesic Effects of Ganoderma Applanatum (Mushroom) Along With Ligand‐Receptor Binding Screening Provide New Insights: Multi‐Disciplinary Approaches.” Biochemistry and Biophysics Reports 27: 101062. 34286108 10.1016/j.bbrep.2021.101062 PMC8278240

[fsn371088-bib-0033] JG, H. 2001. “Drugs and the Treatment of Psychiatric Disorders.” In Goodman & Gilman's the Pharmacological Basis of Therapeutics, 456–470. McGraw‐Hill.

[fsn371088-bib-0034] Jiang, Y. , T. Peng , U. Gaur , et al. 2019. “Role of Corticotropin Releasing Factor in the Neuroimmune Mechanisms of Depression: Examination of Current Pharmaceutical and Herbal Therapies.” Frontiers in Cellular Neuroscience 13: 290. 31312123 10.3389/fncel.2019.00290 PMC6614517

[fsn371088-bib-0035] Jiko, P. A. , M. Mohammad , F. T. Richi , et al. 2024. “Anti‐Inflammatory, Analgesic and Anti‐Oxidant Effects of *Shirakiopsis Indica* (Willd). Fruit Extract: A Mangrove Species in the Field of Inflammation Research.” In Journal of Inflammation Research, 5821–5854. Taylor & Francis. 10.2147/JIR.S470835 PMC11370890 39228677

[fsn371088-bib-0036] JK, P. 2009. “Antihyperlipidemic Acitivity of SYZYGIUM CUMINI Linn. Seed Extract on High Cholesterol Fed Diet Rats.” https://www.doc‐developpement‐durable.org/file/Culture/Arbres‐Bois‐de‐Rapport‐Reforestation/FICHES_ARBRES/Syzygium%20cumini‐jamblon‐jamelonier/SYZYGIUM%20CUMINI%20ANTIHYPERLIPIDEMIC%20ACITIVITY.pdf.

[fsn371088-bib-0037] Kasper, S. 1998. “Social Phobia: The Nature of the Disorder.” Journal of Affective Disorders 50: S3–S9. 9851572 10.1016/s0165-0327(98)00094-9

[fsn371088-bib-0038] Katzung, B. G. , S. B. Masters , and A. J. Trevor . 2004. Basic & Clinical Pharmacology. McGraw‐Hil.

[fsn371088-bib-0039] Kessler, R. C. , W. T. Chiu , O. Demler , and E. E. Walters . 2005. “Prevalence, Severity, and Comorbidity of 12‐Month DSM‐IV Disorders in the National Comorbidity Survey Replication.” Archives of General Psychiatry 62, no. 6: 617–627. 15939839 10.1001/archpsyc.62.6.617 PMC2847357

[fsn371088-bib-0040] Kurumbail, R. G. , A. M. Stevens , J. K. Gierse , et al. 1996. “Structural Basis for Selective Inhibition of Cyclooxygenase‐2 by Anti‐Inflammatory Agents.” Nature 384, no. 6610: 644–648. 8967954 10.1038/384644a0

[fsn371088-bib-0041] Lader, M. , A. Tylee , and J. Donoghue . 2009. “Withdrawing Benzodiazepines in Primary Care.” CNS Drugs 23: 19–34. 19062773 10.2165/0023210-200923010-00002

[fsn371088-bib-0043] Mohammad, M. , I. Anisul , M. Jahirul Islam , et al. 2025. “Methanolic Extract of Edible *Lasia spinosa* Rhizome: A Potential Natural Source of Analgesic, Diuretic, and Thrombolytic Agents.” Journal of Herbs, Spices & Medicinal Plants 31, no. 3: 1–26. 10.1080/10496475.2025.2500533.

[fsn371088-bib-0042] Mohammad, M. , M. T. Chowdhury , N. H. Eshaque , et al. 2025. “Overdose Toxicological Effect of Methanol Extract of Popular Edible *Colocasia esculenta* Linn. Flowers: Biochemical, Hematological, and Behavioral Study on Swiss Albino Mice.” Food Science & Nutrition 13, no. 7: e70674. 10.1002/fsn3.70674. 40703613 PMC12284421

[fsn371088-bib-0044] Mohammad, M. , M. J. I. Mamun , M. Khatun , et al. 2025. “A Multifaceted Exploration of *Shirakiopsis indica* (Willd) Fruit: Insights Into the Neuropharmacological, Antipyretic, Thrombolytic, and Anthelmintic Attributes of a Mangrove Species.” Drugs and Drug Candidates 4, no. 3: 31.

[fsn371088-bib-0045] Mohammad, M. , M. H. Rasel , F. T. Richi , et al. 2025. “Neuropharmacological, Cytotoxic, and Anthelmintic Potentials of *Lasia spinosa* (L.) Thwaites Rhizome: In Vivo, In Vitro, and Computational Approach.” Pharmacological Research—Natural Products 7: 100254. 10.1016/j.prenap.2025.100254.

[fsn371088-bib-0046] Mohanan, N. , and M. Sivadasan . 2002. Flora of Agasthyamala. Bishen Singh Mahendra Pal Singh.

[fsn371088-bib-0047] Mollik, M. A. H. , M. S. Hossan , A. K. Paul , M. Taufiq‐Ur‐Rahman , R. Jahan , and M. Rahmatullah . 2010. “A Comparative Analysis of Medicinal Plants Used by Folk Medicinal Healers in Three Districts of Bangladesh and Inquiry as to Mode of Selection of Medicinal Plants.” Ethnobotany Research and Applications 8: 195–218.

[fsn371088-bib-0048] Morris, G. M. , and M. Lim‐Wilby . 2008. “Molecular Docking.” In Molecular Modeling of Proteins, 365–382. Humana Press. 10.1007/978-1-59745-177-2_19 18446297

[fsn371088-bib-0049] Osman, H. , A. A. Rahim , N. M. Isa , and N. M. Bakhir . 2009. “Antioxidant Activity and Phenolic Content of *Paederia foetida* and *Syzygium aqueum* .” Molecules 14, no. 3: 970–978. 19305354 10.3390/molecules14030970 PMC6253778

[fsn371088-bib-0050] Parasuraman, S. 2011. “Prediction of Activity Spectra for Substances.” Journal of Pharmacy and Pharmacology 2, no. 1: 52–53. 10.4103/0976-500X.77119 PMC3117574 21701651

[fsn371088-bib-0051] Park, M.‐J. , K.‐S. Gwak , I. Yang , et al. 2007. “Antifungal Activities of the Essential Oils in *Syzygium aromaticum* (L.) Merr. Et Perry and *Leptospermum petersonii* Bailey and Their Constituents Against Various Dermatophytes.” Journal of Microbiology 45, no. 5: 460–465. 17978807

[fsn371088-bib-0052] Pires, D. E. V. , T. L. Blundell , and D. B. Ascher . 2015. “pkCSM: Predicting Small‐Molecule Pharmacokinetic and Toxicity Properties Using Graph‐Based Signatures.” Journal of Medicinal Chemistry 58, no. 9: 4066–4072. 25860834 10.1021/acs.jmedchem.5b00104 PMC4434528

[fsn371088-bib-0053] Poroikov, V. , and D. Filimonov . 2005. “PASS: Prediction of Biological Activity Spectra for Substances.” In Predictive Toxicology, 471–490. CRC Press.

[fsn371088-bib-0054] Rahman, H. , M. Rahman , M. Islam , and S. Reza . 2011. “The Importance of Forests to Protect Medicinal Plants: A Case Study of Khadimnagar National Park, Bangladesh.” International Journal of Biodiversity Science, Ecosystem Services & Management 7, no. 4: 283–294.

[fsn371088-bib-0055] Rispin, A. , D. Farrar , E. Margosches , et al. 2002. “Alternative Methods for the Median Lethal Dose (LD50) Test: The Up‐And‐Down Procedure for Acute Oral Toxicity.” ILAR Journal 43, no. 4: 233–243. 12391399 10.1093/ilar.43.4.233

[fsn371088-bib-0056] Saddala, M. S. , P. Jyothi , and A. U. Rani . 2013. “Structure Based Virtual Screening for DNA Methyl Transferase‐1 Inhibitors.” Online Journal of Bioinformatics 14, no. 2: 186–196.

[fsn371088-bib-0057] Santosh, P. , R. Venugopl , A. S. Nilakash , S. Kunjbihari , and L. Mangala . 2011. “Antidepressant Activity of Methanolic Extract of *Passiflora foetida* Leaves in Mice.” International Journal of Pharmacy and Pharmaceutical Sciences 3, no. 1: 112–115.

[fsn371088-bib-0058] Saper, C. B. , and C. D. Breder . 1994. “The Neurologic Basis of Fever.” New England Journal of Medicine 330, no. 26: 1880–1886. 7832832 10.1056/NEJM199406303302609

[fsn371088-bib-0059] Shah, M. S. , M. A. Tayab , A. Rahman , et al. 2022. “Anxiolytic, Antidepressant and Antioxidant Activity of the Methanol Extract of Canarium Resiniferum Leaves.” Journal of Traditional and Complementary Medicine 12, no. 6: 567–574. 36325239 10.1016/j.jtcme.2022.07.001 PMC9618395

[fsn371088-bib-0060] Tareq, A. M. , S. Farhad , A. B. M. N. Uddin , et al. 2020. “Chemical Profiles, Pharmacological Properties, and In Silico Studies Provide New Insights on *Cycas pectinata* .” Heliyon 6, no. 6: e04061. 32529070 10.1016/j.heliyon.2020.e04061 PMC7283161

[fsn371088-bib-0061] Vonshak, A. , O. Barazani , P. Sathiyamoorthy , R. Shalev , D. Vardy , and A. Golan‐Goldhirsh . 2003. “Screening South Indian Medicinal Plants for Antifungal Activity Against Cutaneous Pathogens.” Phytotherapy Research 17, no. 9: 1123–1125. 14595602 10.1002/ptr.1399

[fsn371088-bib-0062] Walf, A. A. , and C. A. Frye . 2007. “The Use of the Elevated Plus Maze as an Assay of Anxiety‐Related Behavior in Rodents.” Nature Protocols 2, no. 2: 322–328. 17406592 10.1038/nprot.2007.44 PMC3623971

[fsn371088-bib-0063] Walter, E. J. , S. Hanna‐Jumma , M. Carraretto , and L. Forni . 2016. “The Pathophysiological Basis and Consequences of Fever.” Critical Care 20: 1–10. 27411542 10.1186/s13054-016-1375-5 PMC4944485

[fsn371088-bib-0064] World Health Organization . 2001. The World Health Report 2001: Mental Health: New Understanding, New Hope. World Health Organization.

[fsn371088-bib-0065] Yankelevitch‐Yahav, R. , M. Franko , A. Huly , and R. Doron . 2015. “The Forced Swim Test as a Model of Depressive‐Like Behavior.” Journal of Visualized Experiments: JoVE 97, no. 52: 587. 10.3791/52587 PMC4401172 25867960

[fsn371088-bib-0066] Zhang, Z.‐J. 2004. “Therapeutic Effects of Herbal Extracts and Constituents in Animal Models of Psychiatric Disorders.” Life Sciences 75, no. 14: 1659–1699. 15268969 10.1016/j.lfs.2004.04.014

[fsn371088-bib-0067] Zishan, S. A. , M. M. Uddin , M. Mohammad , et al. 2023. “In Vivo and In Vitro Initiatives for the Examination of Pharmacological Properties of Brassaiopsis Hainla Leaves.” Asian Journal of Research in Botany 9, no. 1: 15–28.

